# Supplementary material for: Occupational exposure to blood/body fluid splash and its predictors among midwives working in public health institutions at Addis Ababa city Ethiopia, 2020. Institution-based cross-sectional study
Source: PLoS One. 2021 Jun 18;16(6):e0251815. doi: 10.1371/journal.pone.0251815 (PMC8213160; doi:10.1371/journal.pone.0251815)
Supplement: S1 File — (DOCX) [file pone.0251815.s001.docx]

**Questionnaire for health care workers**

Read the questions carefully and **circle** the number among the choices and **write an answer** to open questions in the space provided. You can select **one option**, select **more than one option**, or **give the word/words or numbers** according to the particular questions.

Name of the health institution

Date

Code no of the questionnaire

**Instruction:** circle or make a thick the one you choose from the alternatives from the right side options for the related questions which are placed on the left side.

| **Section one: Socio-demographic** | | | **Skip to** |
| --- | --- | --- | --- |
| 101 | Age |  |  |
| 102 | Gender | 1 Male  2 Female |  |
| 103 | What is your current marital status? | 1. Married 2. Single 3. Separated 4. Divorced 5. Widowed |  |
| 104 | Educational status | 1. Diploma 2. Degree and above |  |
| 106 | What is your average monthly salary? | __________ETB |  |
| 107 | Year of service after graduation | ____________________________________- |  |
| **Section 2. Work-related history** | | |  |
| 201 | Did you work in shifts? | 1. Yes 2. No | If No skip to 203 |
| 202 | If yes to Q #201, which shift? | 1. Day 2. Night |  |
| 203 | On average, how many hours did you worked per week? | 1. ≤39 hours 2. ≥40 hours |  |
| 204 | Is there any infection prevention committee in your Hospital? | 1. Yes 2. No |  |
| 205 | Did you receive training on IP in the last one year? | 1. Yes 2. No |  |
| 206 | Do you use safety containers regularly? | 1. Yes 2. No |  |
| 207 | Do you regularly apply universal precautions? | 1. Yes 2. No |  |
| 208 | In the last one year have you had any needle stick injury? | 1. Yes 2. No | If no skip to 212 |
| 209 | If the answer is yes, what type of injury did you sustain? (two or more response is possible) | 1. Deep injury 2. Slight skin penetration 3. Superficial injury 4. Other (specify) _______________ |  |
| 210 | How many times did you sustain needle stick injury ­?­­ | ___________________time/s |  |
| 211 | How did you sustain the injury? (two or more response is possible) | 1. During recapping 2. By a sudden movement of a patient during injection 3. During needle collection 4. When needle showed up in unexpected places like bed sheets or other places   5.Other (specify)___________ |  |
| 212 | In the last year have you had any injury by sharps? (scissors, blade, etc...) | 1. Yes 2. No | If no skip to 216 |
| 213 | If the answer is yes, what type of injury you sustained? (two or more response possible) | 1. Deep injury 2. Slight skin penetration 3. Superficial injury 4. Other (specify) _________________ |  |
| 214 | How many times did you sustain sharps injuries? | ______________time/s |  |
| 215 | How did you sustain the injury? (two or more response is possible) | 1. During the working procedure 2. During cleaning equipment 3. During sharp collection 4. When sharps show up in unexpected places, like bed sheets or other places 5. Other (specify) _________________ |  |
| 216 | If you sustain needlestick injuries or sharps injuries, how was the health status of the source patient in relation to HIV/AIDS? | 1. Known HIV/AIDS positive 2. Clinically suspected HIV/AIDS 3. Unknown status 4. Known HIV/AIDS negative 5. Other (specify) _________________ |  |
| 217 | In last one year have you been exposed to any blood /body fluid? (Without sharps or needles). | 1. Yes 2. No | If no skip to 221 |
| 218 | What type of exposure (blood/body fluid) did you have? (two or more response is possible | 1. Exposure of blood and body fluids to intact skin 2. Exposure of blood and body fluid to the mucous membrane of broken skin 3. Splash to the eyes 4. Splash to face and mouth |  |
| 219 | How many times such exposure did you have? | _________________time/s |  |
| 220 | If you had blood and body fluid exposure how was the health status of the source patient in relation to HIV/AIDS | 1. Known HIV/AIDs positive 2. Clinically suspected HIV/AIDS 3. Unknown status 4. Known HIV/AIDS negative 5. Other (specify) _____________ |  |
| 221 | If you sustained needlestick/ sharp injuries or exposure of blood and body fluids have you ever reported the injuries to concerning bodies? | 1.yes  2.no |  |
| 222 | Where did the exposure occur? | 1. Delivery 2. Family planning 3. EPI 4. ANC 5. ART 6. Others, Specify__________________ |  |
| 223 | Have you taken any action by yourself after the injury? | 1. Yes 2. No | If no skip to 225 |
| 224 | If yes to Q#223, What did you do immediately after the exposure?  (circle that all apply) | 1. Washing with soap and water 2. Wash with iodine or alcohol solution 3. Get tested for HIV 4. Take post-exposure prophylaxis (PEP) 5. Take tetanus anti-toxoid (TAT) 6. Squeezing to extract more blood 7. Applying pressure to stop bleeding 8. Other specify ___________________ |  |
| 225 | What is the most common disease that may be transmitted through contaminated needle and sharps? (two or more response is possible) | 1.HIV/AIDS  2.HBV(hepatitis B virus)  3.HCV(hepatitis c virus)  4. I don't know  5. other__________________ |  |
| 226 | How do you protect yourself and your patient from infections in the workplace?(two or more response is possible) | 1. Proper hand washing.  2.appropriatly wearing gloves  3.wearing of eye goggles, masks, and shields  4.wearing gowns and aprons  5.other____________________ |  |
| 227 | Have you ever had tested for HIV? | 1.yes  2.no |  |
| 228 | Have you ever had tested for HBV or HCV? | 1.yes  2.no |  |
| **Section 3. Aseptic technique, using of protective barrier and infection prevention** | | |  |
| 301 | Do you wash your hands thoroughly before handling and putting on gloves? | 1. Always 2. Sometimes 3. Never |  |
| 302 | Do you wash your hand after handling objects that might be contaminated? | 1.always  2.sometimes  3.never |  |
| 303 | Do you wash your hand after contact with blood or mucous membrane? | 1.always  2.sometimes  3.never |  |
| 304 | Do you use antiseptic hand rub available before and after contact with each patient? | 1.always  2.sometimes  3.never |  |
| 305 | Do you use antiseptics (e.g., alcohol, savlon, iodine) to remove or kill microorganisms on the instrument or other items? | 1.always  2.sometimes  3.never |  |
| 306 | Do you use protective barriers appropriately? | 1.always  2.sometimes  3.never |  |
| 307 | Are you satisfied with your job? | 1.Satisfied  2.dis satisfied |  |
| 308 | Do you have job-related stress? | 1.Yes  2.No |  |

Thank you very much for taking the time to answer the questions. I appreciate your help, and wish you the best!
