# Supplementary material for: Occupational exposure to blood/body fluid splash and its predictors among midwives working in public health institutions at Addis Ababa city Ethiopia, 2020. Institution-based cross-sectional study
Source: PLoS One. 2021 Jun 18;16(6):e0251815. doi: 10.1371/journal.pone.0251815 (PMC8213160; doi:10.1371/journal.pone.0251815)
Supplement: Attachment — Submitted filename: PONE-D-20-40344_R1_reviewer _Reviewer.pdf [file pone.0251815.s005.pdf]

PLOS ONE

Occupational Exposure to Blood/ Body Fluid Splash and Its Predictors among Midwives Working in Public Health Institutions at Addis Ababa City Ethiopia, 2020

--Manuscript Draft--

**Manuscript Number:**	PONE-D-20-40344R1
**Article Type:**	Research Article
**Full Title:**	Occupational Exposure to Blood/ Body Fluid Splash and Its Predictors among Midwives Working in Public Health Institutions at Addis Ababa City Ethiopia, 2020
**Short Title:**	Occupational Exposure to Blood/ Body Fluid Splash and Its Predictors among Midwives 0
**Corresponding Author:**	Solomon Shitu Ayen, MSc<br>Wolkite University<br>Wolkite, ETHIOPIA
**Keywords:**	Blood/body fluid splash; midwives; Addis Ababa
**Abstract:**	Background: Blood/body fluid splash are hazards to health care professionals in their working area. Around twenty bloodborne pathogens are known to be transmitted through these occupational injuries. This problem alters the health status of health care professionals in different ways, including physically, mentally, and psychologically. Even though health professionals especially midwives who are working at delivery room are highly affected, little known about the exposure. So, this study was aimed to assess the prevalence of exposure to blood/body fluid splash and its predictors among midwives working in public health institutions of Addis Ababa city.<br>Methods: Institution based cross-sectional study was conducted among 438 study participants in public health institutions in Addis Ababa. Data was collected from March 1-20, 2020 by a self-administered questionnaire. The data were entered into Epi data version 3.1 and then exported to SPSS version 24 for analysis. All variables with P<0.25 in the bivariate analysis were included in a final model and statistical significance was declared at P< 0.05.<br>Results: In this study, a total of 424 respondents respond yielding a response rate of 97%. The prevalence of blood and body fluid splashes (BBFs) was 198 (46.7%). Not training on infection prevention, working by two-shifts than three shifts, not regularly apply universal precautions, job-related stress, an average monthly salary of 5001-8000 were independent predictors of blood and body fluid splashes.Conclusion: The study revealed that nearly half of midwives exposed to BBFS. This sparks light to concerned bodies like policy makers and stack holders to design appropriate policy to avert this magnitude and making the environment enabling to comply with standard precautions. We recommend that this study may be done by including rural setting institutions and by including other health professionals that are susceptible for BBFS at work. Formal training on infection prevention and safety practice to apply universal precautions will be needed from the concerned bodies to prevent exposures to blood/body fluid splash.
**Order of Authors:**	Solomon Shitu Ayen, MSc
	Getachew Adugna
	Haimanot Abebe
**Response to Reviewers:**	Author's Point-by-Point Response to the Reviewer's and Editors Reports<br>Title: Occupational Exposure to Blood/ Body Fluid Splash and Its Predictors among Midwives Working in Public Health Institutions at Addis Ababa City Ethiopia, 2020<br>Institution-based cross-sectional study<br>Corresponding author: Solomon Shitu Ayen (solomonsht7@gmail.com)<br>Authors<br>Getachew Adugna (getchadu2013@yahoo.com)<br>Haimanot Abebe (haimanotabebe78@gmail.com)<br>ID: PONE-D-20-40344<br>Journal: PLOS ONE<br>Article type: Research article

Powered by Editorial Manager® and ProduXion Manager® from Aries Systems Corporation

	First of all, the authors would like to thank PLOS ONE Journal editors and the respective reviewers for reviewing our manuscript and providing the necessary comments to be corrected. As per the comments given, we have made corrections point by point to comment. The authors tried to answer all the issues raised by the editorial team and reviewers. Please note that we gave our response in blue font color.<br>Point by point response to editor<br>Comment "Please ensure that your manuscript meets PLOS ONE's style requirements, including those for file naming". And we suggest you thoroughly copyedit your manuscript for language usage, spelling, and grammar<br>Response 1: Thanks for your great suggestion and timely comments. Corrected<br>Point by point response to Reviewer# 1<br>Dear,<br>Question1; Grammatical edits within the document<br>Response 1: Thanks for your great suggestion and timely comments. Corrected grammatical errors throughout the document<br>Question2; Why target this group of health care professionals - not clear from the background<br>Response 2: Thanks for your great suggestion and timely comments. We included only midwives was as in general little is known about blood and body fluid splash among all health professionals and midwives working more focus on conducting delivery in which they are more susceptible for fluids and blood splash during attending delivery. Because they are nearest to exposure like amniotic fluid, meconium, blood, vomiting, and so on.<br>Question3; Rewrite the conclusion<br>Response 3: Thanks for your great suggestion and timely comments. Corrected as, "The study revealed that nearly half of midwives exposed to BBFS. This sparks light to concerned bodies like policymakers and stack holders to design appropriate policy to avert this magnitude and making the environment enabling to comply with standard precautions. Also, this study was done in urban health settings, and the institutions are better equipped with personal protective materials than the rural settings. So, we recommend that this study may be done by including rural setting institutions and by including other health professionals that are susceptible to BBFS at work. Formal training on infection prevention and safety practice to apply universal precautions will be needed from the concerned bodies to prevent exposures to blood/body fluid splash."<br>Question 4; Rewrite the background<br>Response 4: Thanks for your great suggestion and timely comments. Corrected as Blood/body fluid splash (BBFS) are hazards to health care workers in their working area. Around twenty bloodborne pathogens are known to transmit through this occupational exposure [1]. This problem alters the health status of health care professionals (HCPs) in different ways, including physically, mentally, and psychologically [2].<br>No occupation or work is free from health hazards. People are always under a certain amount of health risk wherever they are working [3]. Health care professionals are exposed to a wide range of occupational health and safety hazards [4]. An estimated 20 million work-related injuries and 390, 000 new work-related illnesses occur each year in the United States and China 66.3% of HCPs per year exposed to BBFS; however, the number of occupational diseases and injuries reported each year is much more [4, 5].<br>Nowadays, workplace injuries, especially BBFS prevalence, are increasing among health workers [6, 7]. Occupational exposure to BBFS in healthcare facilities constitutes a significant risk of transmission of human immune deficiency virus (HIV) and other blood-borne pathogens to healthcare workers [8-10]<br>As estimated by the Center for Disease Control (CDC) that every year more than three million HCPs are exposed to BBFs, through sharps, plus mucocutaneous injuries in the United States [10]. Due to BBFS, the possibility of infections ranges from as low as 0.2–0.5% for HIV to as huge as 3–10% for Hepatitis C virus (HCV) and 40% for Hepatitis B virus (HBV) [11]. World health organization (WHO) estimates that nearly 3 million HCPs experience occupational exposure to blood-borne viruses HBV, HCV, and HIV has been reported to be 2.1 million, 926,000, and 327,000 respectively [12-15]. From this 90% of this exposure to infection was resulted from low resource countries [14]. In Ethiopia, studies indicated that the prevalence of HCV due to BBFS exposure was 0.9- 5.8% [3, 16].<br>Health care workers in Africa experience from two to four needle-stick injuries per year

on average, with Nigeria, Tanzania, and South Africa reporting on average of 2.10 injuries per HCPs per year [17].
Even though there is no national data regarding the prevalence of BBFS among midwives in Ethiopia previous studies in Eastern, Southern, and Northern parts have shown the increased risk of occupational blood exposure [17-19]. The majority of these studies were only confined to a single institution and workplace [18, 19]. The high rate of HCPs is affected by the exposure [2]. Despite the presence of many possibilities to reduce the burden of occupational exposure to BBFS, it is not yet recognized. Hence this research was carried out on midwives working at public health institutions of Addis Ababa city delivery unit due to they are more exposed and the first line for BBFS.
Question 5; why you focus only on midwives?
Response 5: Thanks for your great suggestion and timely comments. Even though all health professionals are exposed to BBFS, midwives are the first line for fluid and blood exposure. Because obstetrics is a bloody profession and they are the nearby professional. And all health institutions have employed midwives in Addis Ababa city.
Question 6; Study Population
Randomly selected midwives working in public health institutions of Addis Ababa city. "How was this done?"
Response 6: Thanks for your great suggestion and timely comments. By using a systematic random sampling technique
Question 7; On sampling procedure, we said The registers of all midwives were obtained from the administration of the offices. "Obtaining this list of names does pose some ethical considerations. Need to clarify how the researchers obtained access to this register. Had the midwives provided informed consent for their names and contact details to be released for research purposes?"
Response 7: Thanks for your great suggestion and timely comments. Even though the study has some ethical issues we directly requested the office by letter but because the study has minimal or no harm on them.
Question 8; more detail of process needed on sampling procedure
Response 8: Thanks for your great suggestion and timely comments. Possible correction taken by each institution has their number of allocated midwives based on that we classify proportionally based on total sample size. "Six public hospitals from 12 hospitals and 66 health centers from 112 health centers were selected by simple random sampling technique. The total number of midwives working in Addis Ababa city was 2432 and the number of midwives working in selected public health institutions was 1296. To select study participants from selected health institutions the proportional allocation method (each institution's number of midwives was allocated by proportion) was used for each institution. Study participants who fulfilled the inclusion criteria were selected by systematic random sampling technique until the required number is obtained with a kth value of 3."
Question 9; Data quality control and collection methods
Using an English version questionnaire "is this a limitation? English literacy levels etc"
Response 9: Thanks for your great suggestion and timely comments. This study was related to their job and doesn't need that much fluent speaker or writer of English. The interviewees were minimum of diploma certificate so they can understand the questionnaire.
Question 10; in result part "is this reflective of the midwifery workforce demographic in Ethiopia? If not then why more males completed"
Response 10: Thanks for your great suggestion and timely comments. Your concern was correct midwifery profession was perceived as a profession of women in the previous era but in the current situation this is changed and more males are involved as midwife professionals. That may be the reason that in this study the percentage of males was greater than females.
Question 11; These data are not presented in a table - sentence structure needs review to make results clear. Also review terminology around HIV/AIDS - was 12.5 living with HIV and been diagnosed with an AIDS-defining illness or is it more accurate to say 12.5% were HIV positive or known to be living with HIV.
Response 11: Thanks for your great suggestion and timely comments. Corrected
Question 12; the meaning of working in shifts is unclear - please clarify
Response 12: Thanks for your great suggestion and timely comments. Corrected as working in two shifts >12 hours work or day and night is more likely exposed than working in only one shift (working for ≤12 hours)

Point by point response to Reviewer# 2

Powered by Editorial Manager® and ProduXion Manager® from Aries Systems Corporation

Dear, Yalemzewod Gelaw (PhD)
Question1; I would like to suggest the authors include a justification why they are interested only in midwives and how the risk of exposure to blood/body fluid splash is varied across health professionals supported with prevalence figures. In the context of the Ethiopian health system, all health facilities have no midwives. Therefore, still, other health professionals such as nurses/public health officers/doctors are at risk for exposure.
Response 1: Thanks for your great suggestion and timely comments. We included only midwives was as in general little is known about blood and body fluid splash among all health professionals and midwives working more focus on conducting delivery in which they are more susceptible for fluids and blood splash during attending delivery. Because they are nearest to exposure like amniotic fluid, meconium, blood, vomiting, and so on. Since the study was done in Addis Ababa city the work of the delivery unit was covered by midwives in all health institutions even if the number of professionals is not equal to the standard.
Question 2; "Study setting – the risk of exposure could be high in health professionals working in rural settings than in health professionals working in urban. It would be great if the authors could consider the effect of health facilities setting".
Response 2: Thanks for your great suggestion and timely comments. This is a correct issue that the study was better sound than this if it included the rural setting but due to different reasons, it was done only in the urban area even though little was known on the topic. We recommend other researchers incorporate rural settings in future studies.
Question 3; Source Population – does the result will be generalized only to those midwives working in AA? "All midwives working in public health institutions of Addis Ababa city'' – is the population where your sample was drawn i.e., study population unless the authors' hypothesis was only for urban midwives.
Response 3: Thanks for your great suggestion and timely comments. Yes, this study was generalized for urban setting midwives. Because the source population was midwives working in public health institutions of Addis Ababa city
Question 4; Inclusion criteria – is there unregistered midwives working in the current Ethiopian health system? I would suggest the authors modify this.
Response 4: Thanks for your great suggestion and timely comments. Corrected as "All midwives working in Addis Ababa city"

Question 5; Sample size determination – I would recommend the authors to include the sample size assumptions briefly such as proportion from the previous study, margin of error, etc.
Response 5: Thanks for your great suggestion and timely comments. Corrected as "A separate sample size was calculated for each specific objective by using both single and double population proportion formula was used. For the first specific objective, (To determine the prevalence of occupational exposure to BBFS among midwives working in Addis Ababa city) single population proportion was used.
n= (Z α/2)2 P (1-P) by the assumption of n= minimum sample size required for the study
d2
(Z α/2)2 = standard normal distribution with 95% confidence interval, P= 21% (prevalence of occupational exposure and behavior of health care workers in Ethiopia) [18], d= is a tolerable margin of error (d=0.04)
The sample size of the first objective was greater than that of the second objective. By adding a 10% non-response rate from the largest sample size the calculated sample size was 438."
Question 6; Sampling procedures – "…. simple random sampling technique until the required number is obtained." For simple random sampling techniques, all eligible study participants should be identified before the data collection starts. Including study participants, until the required number is obtained will work for systematic sampling techniques.
Response 6: Thanks for your great suggestion and timely comments. Corrected as "The total number of midwives working in Addis Ababa city public health institutions was 2432 and number of midwives working in selected institutions was 1296. To select study participants from selected health institutions the proportional allocation method (each institution's number of midwives was allocated by proportion) was used. Study participants who fulfilled the inclusion criteria were selected by systematic random sampling technique until the required number is obtained with a kth value of 3".
Question 7; The independent variables except the demographic variables need to be

	clearly defined.<br>Response 7: Thanks for your great suggestion and timely comments. Corrected as Work-related factors: (Training on IP, working environment, year of service, educational level)<br>Behavioral factors: (Job satisfaction, reporting after the exposure, using the universal precautions and personal protective equipment)<br>Question 8; I would like to ask the authors to use variables with the (P-value < 0.25) to include in the multivariable analysis. For the authors' reference – to read applied logistic regression book by Hosmer and Lemeshow.<br>Response 8: Thanks for your great suggestion and timely comments. Corrected as "The collected data were checked and reviewed for completeness, coded, and entered into Epi data version 3.1 and exported to SPSS version 24 for analysis. Descriptive statistics were used to determine the frequency of different variables. Association between dependent and independent variables were examined using bi-variable and multivariable logistic regression models. Those variables which were (P-value < 0.25) [18] in the bivariate analysis were included in the final model of multivariate analysis to control all possible confounders. Multi-collinearity was checked to see the linear correlation among the independent variables by using standard error. Variables with a standard error of > 2 were dropped from the multivariable analysis. Model fitness was checked with the Hosmer-Lemeshow test. The direction and strength of statistical association were measured by the odds ratio with 95 % CI using multivariable logistic regression analysis. Adjusted odds ratio along with 95% CI was estimated to identify factors associated with BBFS. In this study P-value < 0.05 was considered to declare a result as statistically significant".<br>Question 9; Socio-demographic characteristics – report median age instead of the mean. The min and max age showed the distribution is not normal.<br>Response 9: Thanks for your great suggestion and timely comments. Corrected as "The age distribution of the respondents ranges from 20-56 years, with a mean and median age of 30.11 (SD ± 5.37), 26.5 years respectively and 219 (51.7%) were between the age group of 25-29 years".<br>Question 10; Regression output – interpret only the multivariable results.<br>Response 10: Thanks for your great suggestion and timely comments. Corrected as "In multivariable logistic regression working in >12 hours shift, not receive IP and safety practice training, not regularly apply universal precautions, job-related stress, an average monthly salary of 5001-8000 were found independent predictors of BBFS. Health care professionals who did not regularly apply universal precautions were 8 times more likely to have blood/body fluid splash (AOR 8, 95% CI 6.55 - 14.8) than their counterparts. Participants who did not receive IP and safety practice training were four times more likely to have blood/body fluid splash (AOR 4.27, 95% CI 1.94 - 9.41) than those who did receive training. The odds ratio was two times higher for those working in two shifts (more than 12 hours) (AOR 2.36, 95% CI 1.034-5.4) than one shift (≤ 12 hours). The odds of exposure to BBFs were two times increased for those who had job-related stress (AOR 2.18, 95% CI 1.07-4.49) than their counterpart. Respondents who get an average monthly salary of 5001-8000 Ethiopian birr were 70% less likely to be exposed to BBFS (AOR 0.305, 95% CI 0.105 - 0.88) than those who had gained less than 8000 Ethiopian birrs.<br>Question 11; Discussion section is very weak and needs to be improved.<br>Response 11: Thanks for your great suggestion and timely comments. Rewritten and improved as much as possible
**Additional Information:**	
**Question**	**Response**
**Financial Disclosure**<br><br>Enter a financial disclosure statement that describes the sources of funding for the work included in this submission. Review the submission guidelines for detailed requirements. View published research articles from *PLOS ONE* for specific examples.	The author(s) received no specific funding for this work

This statement is required for submission and **will appear in the published article** if the submission is accepted. Please make sure it is accurate.<br><br>**Unfunded studies**<br>Enter: *The author(s) received no specific funding for this work.*<br><br>**Funded studies**<br>Enter a statement with the following details:<br>• Initials of the authors who received each award<br>• Grant numbers awarded to each author<br>• The full name of each funder<br>• URL of each funder website<br>• Did the sponsors or funders play any role in the study design, data collection and analysis, decision to publish, or preparation of the manuscript?<br>• **NO** - Include this sentence at the end of your statement: *The funders had no role in study design, data collection and analysis, decision to publish, or preparation of the manuscript.*<br>• **YES** - Specify the role(s) played.<br><br>* typeset	
**Competing Interests**<br><br>Use the instructions below to enter a competing interest statement for this submission. On behalf of all authors, disclose any competing interests that could be perceived to bias this work—acknowledging all financial support and any other relevant financial or non-financial competing interests.<br><br>This statement **will appear in the published article** if the submission is accepted. Please make sure it is accurate. View published research articles from *PLOS ONE* for specific examples.	The authors have declared that no competing interests exist.

**NO authors have competing interests**<br><br>Enter: *The authors have declared that no competing interests exist.*<br><br>**Authors with competing interests**<br><br>Enter competing interest details beginning with this statement:<br><br>*I have read the journal's policy and the authors of this manuscript have the following competing interests: [insert competing interests here]*<br><br>* typeset	
**Ethics Statement**<br><br>Enter an ethics statement for this submission. This statement is required if the study involved:<br><br>• Human participants<br>• Human specimens or tissue<br>• Vertebrate animals or cephalopods<br>• Vertebrate embryos or tissues<br>• Field research<br><br>Write "N/A" if the submission does not require an ethics statement.<br><br>General guidance is provided below. Consult the submission guidelines for detailed instructions. **Make sure that all information entered here is included in the Methods section of the manuscript.**	Ethical clearances were obtained from institutional review boards (IRB) of Addis Ababa University. An official letter of permission was submitted to the Addis Ababa city health bureau.<br>A formal letter was submitted to selected public health institutions from the bureau. The respondents were informed about the objective, purpose, risks, and benefits of the study and the<br>right to refuse to participate, and then informed written and signed consent was taken. The study posed a low or no more than minimal risk to the study participants. Also, the study did<br>not involve any invasive procedures. Moreover, the confidentiality of information was guaranteed by using code numbers rather than personal identifiers and by keeping the data<br>locked.

Powered by Editorial Manager® and ProduXion Manager® from Aries Systems Corporation

**Format for specific study types**<br><br>**Human Subject Research (involving human participants and/or tissue)**<br>• Give the name of the institutional review board or ethics committee that approved the study<br>• Include the approval number and/or a statement indicating approval of this research<br>• Indicate the form of consent obtained (written/oral) or the reason that consent was not obtained (e.g. the data were analyzed anonymously)<br><br>**Animal Research (involving vertebrate animals, embryos or tissues)**<br>• Provide the name of the Institutional Animal Care and Use Committee (IACUC) or other relevant ethics board that reviewed the study protocol, and indicate whether they approved this research or granted a formal waiver of ethical approval<br>• Include an approval number if one was obtained<br>• If the study involved *non-human primates*, add *additional details* about animal welfare and steps taken to ameliorate suffering<br>• If anesthesia, euthanasia, or any kind of animal sacrifice is part of the study, include briefly which substances and/or methods were applied<br><br>**Field Research**<br><br>Include the following details if this study involves the collection of plant, animal, or other materials from a natural setting:<br>• Field permit number<br>• Name of the institution or relevant body that granted permission	
**Data Availability**<br><br>Authors are required to make all data underlying the findings described fully available, without restriction, and from the time of publication. PLOS allows rare exceptions to address legal and ethical concerns. See the PLOS Data Policy and FAQ for detailed information.	No - some restrictions will apply

A Data Availability Statement describing where the data can be found is required at submission. Your answers to this question constitute the Data Availability Statement and **will be published in the article**, if accepted.<br><br>**Important:** Stating 'data available on request from the author' is not sufficient. If your data are only available upon request, select 'No' for the first question and explain your exceptional situation in the text box.<br><br>Do the authors confirm that all data underlying the findings described in their manuscript are fully available without restriction?	
**Describe where the data may be found in full sentences. If you are copying our sample text, replace any instances of XXX with the appropriate details.**<br><br>• If the data are **held or will be held in a public repository**, include URLs, accession numbers or DOIs. If this information will only be available after acceptance, indicate this by ticking the box below. For example: *All XXX files are available from the XXX database (accession number(s) XXX, XXX.*).<br>• If the data are all contained **within the manuscript and/or Supporting Information files**, enter the following: *All relevant data are within the manuscript and its Supporting Information files.*<br>• If neither of these applies but you are able to provide **details of access elsewhere**, with or without limitations, please do so. For example:<br><br>*Data cannot be shared publicly because of [XXX]. Data are available from the XXX Institutional Data Access / Ethics Committee (contact via XXX) for researchers who meet the criteria for access to confidential data.*<br><br>*The data underlying the results presented in the study are available from (include the name of the third party*	All relevant data are within the manuscript and its Supporting Information files

and contact information or URL).<br>• This text is appropriate if the data are owned by a third party and authors do not have permission to share the data.<br><br>* typeset	
Additional data availability information:	

Powered by Editorial Manager® and ProduXion Manager® from Aries Systems Corporation

Manuscript Click here to access/download;Manuscript;mnuscript.docx

Occupational exposure to blood/ body fluid splash and its predictors among midwives working in public health institutions at Addis Ababa city Ethiopia, 2020

Institution-based cross-sectional study

[1]Solomon Shitu *, [2]Getachew Adugna, [3]Haimanot Abebe

[1]Department of Midwifery, Wolkite University College of Health and Medical Sciences, Wolkite University, Wolkite, Ethiopia

[2] City Health Offices, Addis Ababa City Health Departments, Addis Ababa, Ethiopia

[3] Department of Public Health, Wolkite University College of Health and Medical Sciences, Wolkite University, Wolkite, Ethiopia

Email address: [1]solomonsht7@gmail.com,[2]getchadu2013@yahoo.com, [3]haimanotabebe78@gmail.com

Educational qualifications: [1] Lecturer (BScMw, MSc)

[2, 3] Lecturer (BSc public health, Mph)

*Corresponding Author

Corresponding Author Address

Email address: solomonsht7@gmail.com

Cell phone +251924704053

Postal address 07 Wolkite, Ethiopia

Living address wolkite town

Abstract

Background: Blood/body fluid splash are hazards to health care professionals in their working area. Around twenty bloodborne pathogens are known to be transmitted through these occupational injuries. This problem alters the health status of health care professionals in different ways, including physically, mentally, and psychologically. Even though health professionals especially midwives who are working in delivery rooms are highly affected, little is known about the exposure. So, this study was aimed to assess the prevalence of exposure to blood/body fluid splash and its predictors among midwives working in public health institutions of Addis Ababa city.

Methods: Institution based cross-sectional study was conducted among 438 study participants in public health institutions in Addis Ababa. Data was collected from March 1-20, 2020 by a self-administered questionnaire. The data were entered into Epi data version 3.1 and then exported to SPSS version 24 for analysis. All variables with P<0.25 in the bivariate analysis were included in a final model and statistical significance was declared at P< 0.05.

Results: In this study, a total of 424 respondents respond yielding a response rate of 97%. The prevalence of blood and body fluid splashes (BBFs) was 198 (46.7%). Not training on infection prevention, working in two shifts (> 12 hours), not regularly apply universal precautions, job-related stress, an average monthly salary of 5001-8000 were independent predictors of blood and body fluid splashes.

Conclusion: The study revealed that nearly half of midwives were exposed to BBFS. This sparks light to concerned bodies like policymakers and stack holders to design appropriate policy to avert this magnitude and making the environment enabling to comply with standard precautions. We recommend that this study may be done by including rural setting institutions and by including other health professionals that are susceptible to BBFS at work. Formal training on infection prevention and safety practice to apply universal precautions will be needed from the concerned bodies to prevent exposures to blood/body fluid splash.

Keywords: Blood/body fluid splash, midwives, Addis Ababa

Introduction

Blood/body fluid splash (BBFS) are hazards to health care workers in their working area. Around twenty bloodborne pathogens are known to transmit through this occupational exposure [1]. This problem alters the health status of health care professionals (HCPs) in different ways, including physically, mentally, and psychologically [2].

No occupation or work is free from health hazards. People are always under a certain amount of health risk wherever they are working [3]. Health care professionals are exposed to a wide range of occupational health and safety hazards [4]. An estimated 20 million work-related injuries and 390, 000 new work-related illnesses occur each year in the United States and China 66.3% of HCPs per year exposed to BBFS; however, the number of occupational diseases and injuries reported each year is much more [4, 5].

Nowadays, workplace injuries, especially BBFS prevalence, are increasing among health workers [6, 7]. Occupational exposure to BBFS in healthcare facilities constitutes a significant risk of transmission of human immune deficiency virus (HIV) and other blood-borne pathogens to healthcare workers [8-10]

As estimated by the Center for Disease Control (CDC) that every year more than three million HCPs are exposed to BBFs, through sharps, plus mucocutaneous injuries in the United States [10]. Due to BBFS, the possibility of infections ranges from as low as 0.2–0.5% for HIV to as huge as 3–10% for Hepatitis C virus (HCV) and 40% for Hepatitis B virus (HBV) [11]. World health organization (WHO) estimates that nearly 3 million HCPs experience occupational exposure to blood-borne viruses HBV, HCV, and HIV has been reported to be 2.1 million, 926,000, and 327,000 respectively [12-15]. From this 90% of this exposure to infection was resulted from low resource countries [14]. In Ethiopia, studies indicated that the prevalence of HCV due to BBFS exposure was 0.9- 5.8% [3, 16].

Health care workers in Africa experience from two to four needle-stick injuries per year on average, with Nigeria, Tanzania, and South Africa reporting on average of 2.10 injuries per HCPs per year [17].

Even though there is no national data regarding the prevalence of BBFS among midwives in Ethiopia previous studies in the Eastern part of the country showed that the prevalence of BBFS

was 28.8% [18]. The majority of these studies were only confined to a single institution and workplace [18, 19]. A high rate of midwives is affected by the exposure [2]. Despite the presence of many possibilities to reduce the burden of occupational exposure to BBFS, it is not yet recognized. Hence this research was carried out on midwives working at public health institutions of Addis Ababa city due to they are more exposed and the first line for BBFS.

Methods

Study design, area, and period

An institution-based cross-sectional study was conducted from March 1-20, 2020 in selected public health institutions of Addis Ababa Ethiopia's capital city. It comprises 10 sub-cities. In the City, there are 12 public hospitals and 112 health centers. All institutions serve the population of Addis Ababa and provide all services including maternal and child health care service [42].

Population

Source population

All midwives working in public health institutions of Addis Ababa city.

Study population

Randomly selected midwives working in public health institutions of Addis Ababa city.

Inclusion criteria

All midwives working in Addis Ababa city

Exclusion criteria

Midwives who were on annual or maternity leave and who were critically ill and unable to respond during data collection.

Sample size determination

Separate sample size was calculated for each specific objective by using both single and double population proportion formula was used. For the first specific objective, (To determine the prevalence of occupational exposure to BBFS among midwives working in Addis Ababa city) single population proportion was used.

$$n = \frac{(Z\,\alpha/2)^2\, P\,(1-P)}{d^2}$$

by the assumption of n= minimum sample size required for the study

$(Z\ \alpha/2)^2$ = standard normal distribution with 95% confidence interval, P= 21% (prevalence of occupational exposure and behavior of health care workers in Ethiopia) [18], d= is a tolerable margin of error (d=0.04)

The sample size of the first objective was greater than that of the second objective. By adding a 10% non-response rate from the largest sample size the calculated sample size was 438.

Sampling procedure

Six public hospitals from 12 hospitals and 66 health centers from 112 health centers were selected by simple random sampling technique. The total number of midwives working in Addis Ababa city public health institutions was 2432 and the number of midwives working in selected public health institutions was 1296. To select study participants from selected health institutions the proportional allocation method (each institution's number of midwives was allocated by proportion) was used for each institution. Study participants who fulfilled the inclusion criteria were selected by systematic random sampling technique until the required number is obtained with a kth value of 3. The registers of all midwives were obtained from the administration of the offices. Any unwilling or unavailable selected participant was replaced by the very next person on the list who had not been already selected to participate.

Dependent variables

Exposure to blood/body fluid splash

Independent variables

Socio-demographic factors: (Age, Sex, Marital status, Qualifications of midwives, Monthly income)

Work-related factors: (Training on IP, working environment, year of service, educational level)

Behavioral factors: (Job satisfaction, reporting after the exposure, using the universal precautions and personal protective equipment)

Data quality control and collection methods

The questionnaire was developed by reviewing different pieces of literature [2, 3, 7, 17, 18]. Data were collected by facilitator guided structured self-administered English version questionnaire. Facilitators took two days of training to assure quality. The questionnaire was checked for completeness before data entry into the software. Proper coding and categorization of data were maintained for the quality of the data to be analyzed. Double data entry was done for its validity and compare to the original data.

Data processing and analysis

The collected data were checked and reviewed for completeness, coded, and entered into Epi data version 3.1, and exported to SPSS version 24 for analysis. Descriptive statistics were used to determine the frequency of different variables. Association between dependent and independent variables were examined using bi-variable and multivariable logistic regression models. Those variables which were (P-value < 0.25) [18] in the bivariate analysis were included in the final model of multivariate analysis to control all possible confounders. Multi-collinearity was checked to see the linear correlation among the independent variables by using standard error. Variables with a standard error of > 2 were dropped from the multivariable analysis. Model fitness was checked with the Hosmer-Lemeshow test. The direction and strength of statistical association were measured by the odds ratio with 95 % CI using multivariable logistic regression analysis. Adjusted odds ratio along with 95% CI was estimated to identify factors associated with BBFS. In this study P-value < 0.05 was considered to declare a result as statistically significant.

Operational Definitions

Blood/Body exposure: those exposed to blood, vomits, urine, sputum, saliva, amniotic fluid, exudative fluids [31].

Personal protective equipment: barriers and filters between the worker and the hazard [1].

Ethical consideration and consent to participate

Ethical clearances were obtained from institutional review boards (IRB) of Addis Ababa University. An official letter of permission was submitted to the Addis Ababa city health bureau. A formal letter was submitted to selected public health institutions from the bureau. The respondents were informed about the objective, purpose, risks, and benefits of the study and the right to refuse to participate, and then informed written and signed consent was taken.

The study posed a low or no more than minimal risk to the study participants. Also, the study did not involve any invasive procedures. Moreover, the confidentiality of information was guaranteed by using code numbers rather than personal identifiers and by keeping the data locked.

Results

Socio-demographic characteristics

A total of 424 respondents completed the questionnaire yielding a response rate of 97%. The age distribution of the respondents ranges from 20-56 years, with a mean and median age of 30.11 (SD ± 5.37), 26.5 years respectively, and 219 (51.7%) were between the age group of 25-29 years. Nearly two-thirds of participants, 258 (60.8%) were males. Unmarried accounted for 253(59.7%). In this study majority, four hundred and ten (96.7%) participants were holders of degrees and above. More than four out of five earned a monthly salary of ≤8000 Ethiopian birrs. Two-third, 295 (69.6%), had less than five years' work experience (Table 1).

Behavior, knowledge, and practice of midwives

Out of the respondents, less than half, 191(45%) responded that they had followed universal precautions regularly, and 187 (44.1%) usually use safety deposit boxes to discard sharp

materials. Concerning the protection of oneself and the patient from infection, more than three fourth 362 (85.4%) of respondents indicated that they wear gloves appropriately. The wearing of gowns and aprons had responded by 300(70.8%). Among study participants, 195 (46%) had responded that they had job-related stress, and 246 (58%) answered that they were dissatisfied with their job. More than two-thirds of 294 (69.3%) of participants reported that they had always washed their hands immediately after handling contaminated objects while 246 (58.5%) always wash their hands before putting on gloves. Three hundred forty-seven 347 (81.8%) reported that they washed their hands always after contact with blood or mucous membrane (Table 2).

Prevalence of blood/body fluid splashes

From a total of 424 midwives who participated in the study occupational exposure to blood/body fluid splash at least once was 198 (46.7%) with 95%CI: 41.3-52.2. Among those exposed to BBFS in the last year, 163(82.3%) of them encountered one to two times, and 14 (7.1%) faced five and more times. The status of the source of exposure was unknown for 79 (39.5%), whereas, 25(12.5%) were known HIV positive, 16 (8%) were clinically suspected HIV positive, however, 77(38.5%) known HIV negative. Only, 3 (1.5%) of the source of exposure was reported unknown hepatitis B positive. Of those BBFS, 138 (60.8%) exposure was encountered to skin. The splash to the broken membrane of the skin, eyes, and mouth were 16 (7.9%), 51 (25.6%), 11 (5.7%) respectively.

Predictors of blood/body fluid splash

According to multivariable logistic regression working in two shifts(working for > 12 hours), not received IP and safety practice training, not regularly apply universal precautions, job-related stress, an average monthly salary of 5001-8000 were independent predictors of BBFS.

Health care professionals who did not regularly apply universal precautions were 8 times more likely to have blood/body fluid splash (AOR 8, 95% CI 6.55 - 14.8) than their counterparts. Participants who did not receive IP and safety practice training were four times more likely to have blood/body fluid splash (AOR 4.27, 95% CI 1.94 - 9.41) than those who did receive training. The odds ratio was two times higher for those working in two shifts (more than 12 hours) (AOR 2.36, 95% CI 1.034-5.4) than one shift (≤ 12 hours). The odds of exposure to BBFs

were two times increased for those who had job-related stress (AOR 2.18, 95% CI 1.07-4.49) than their counterpart. Respondents who get an average monthly salary of 5001-8000 Ethiopian birr were 70% less likely to be exposed to BBFS (AOR 0.305, 95% CI 0.105 - 0.88) than those who had gained less than 8000 Ethiopian birrs (Table 3).

Discussions

In this study, findings indicate that the prevalence of BBFS was 198 (46.7%) with 95%CI: 41.3-52.2 exposed at least once in their work life. The prevalence was in line with the study done in Australia 42% [20]. But it was significantly higher than some findings in Eastern Ethiopia (20.2% and elsewhere 36.1%) [18, 44]. In contrast, this proportion remains low compared to the study done in Gonder and Tigray Ethiopia at 62.9%, 56.3% respectively [2, 19]. The reason for this difference could be methodological differences and variation in study area and periods, and under-reporting in this study. And also may be due to the difference in and the availability of resources. Thus, the study was done in an urban area the availability of materials to apply safety precautions was better than in rural settings. Work cultures may be significantly affecting the outcome the study included only midwives in whose work area is more susceptible for BBFS.

The most potential determinant factor associated with BBFS was not regularly applying universal precaution (AOR 8.43, 95% CI 6.55-14.8). Those who did not apply universal precautions were 8 times more exposed to BBFS than those who apply. This is consistent with studies done in Mongolia [33] and Northwest Ethiopia [38]. This might be because those who did not adhere to universal precautions were more likely to incur occupational exposure due to they are working unprotected.

In this study, midwives who worked in two shifts (≥12 hours) were twice more likely to be exposed to BBFS (AOR 2.36, 95% CI 1.034-5.4) than those who work in only one shift (≤12 hours). This finding was supported by the study done in Germany [14], and Ondo state, Nigeria [34]. Since two-shift work has a high clinical activity schedule, with full of workload and they will stay more than 12 hours in the workplace, so as time elapses they become tired and negligent to apply universal precautions.

The result of the study indicates that lack of training on IP and safety practice in the last year was a significant determinant to BBFS (AOR 4.27, 95% CI 1.94-9.41). This may be because training

enhances or contributed to change the knowledge attitude and practice of individuals. Similarly, a study carried out in Debre Birhan Ethiopia and Kenya [24, 26] revealed that the absence of onsite training on IP and safety practice was the main contributor to occupational exposure. Also maybe knowledge and practice acquired during training necessarily translate into practice on prevention measures and safety precautions.

Midwives who had job-related stress were two times more likely to be exposed to BBFS (AOR 2.18, 95% CI 1.07-4.49) than those who did not have job-related stress. This is in agreement with the study done in Northwest Ethiopia [38]. This may be justified since those who had job-related stress could not focus on their jobs and they may be predisposed to BBFS. And also stress may lead to a lack of concentration on work.

Respondents who earned a monthly salary of 5000-8000 birr were 70% less likely to be exposed to BBFS (AOR 0.30, 95% CI 0.105-0.88) when compared to respondents who got a monthly salary of ≤5000. Possibly may be due to their satisfaction with their monthly salary, they might be involved seriously in their activities. Also, those who earned ≤5000 might be those with newly deployed to work so they may be less experienced and training could be given first for those senior staffs.

Conclusion

The study revealed that nearly half of midwives were exposed to BBFS. This sparks light to concerned bodies like policymakers and stack holders to design appropriate policy to avert this magnitude and making the environment enabling to comply with standard precautions. Also, this study was done in urban health settings, and the institutions are better equipped with personal protective materials than the rural settings. So, we recommend that this study may be done by including rural setting institutions and by including other health professionals that are susceptible to BBFS at work. Formal training on infection prevention and safety practice to apply universal precautions will be needed from the concerned bodies to prevent exposures to blood/body fluid splash.

Strength and limitation of the study

Strength of study

This study provides information about the burden of occupational exposure related to BBFs. It is an unsearched/ neglected thematic area of the health care system and it adds value for the system and serves as a baseline for researchers.

Limitation of study

Information was obtained by asking the respondents, so this may incur recall bias. And due to the self-administered questionnaire, it was susceptible to social desirability bias HCPs might report socially acceptable responses than their actual day-to-day practice that may underestimate the prevalence of the outcome.

The study ~~was~~ included only urban setting institutions and midwives working there so it lacks generalizability ~~in[illegible]ling~~ midwives working in the rural area. And the study has done only on midwives so better to include other health professionals.

Because of the cross-sectional study design, the study could not answer the direction of causality between independent factors and outcome.

Abbreviations

AIDS Acquired Immune Deficiency Syndrome

AOR Adjusted Odds Ratio

BBFS Blood/Body Fluid splash

CDC Center for Disease Control

CI Confidence Interval

COR Crude Odds Ratio

FMoH Federal Ministry of Health

HBV Hepatitis B virus

HCV Hepatitis C virus

HCPs Health Care Providers

HIV Human Immunodeficiency Virus

IP Infection Prevention

OR Odds Ratio

PEP Post Exposure Prophylaxis

SPSS Statistical Package for the Social Sciences

WHO World Health Organization

Declarations

The content of the study is solely the responsibility of the authors.

Consent for publication

Not applicable

Availability of data and materials

The datasets used and/or analyzed during the current study are available from the corresponding author on reasonable request.

Competing interests

The authors would like to declare that we have no competing interest in this study.

Funding

There is no source of funding for this research. All costs were covered by researchers.

Authors’ contributions

All authors made a significant contribution to the work reported, whether that is in the conception, study design, execution, acquisition of data, analysis, and interpretation, or in all these areas; took part in drafting, revising, or critically reviewing the article; gave final approval of the version to be published; have agreed on the journal to which the article has been submitted; and agree to be accountable for all aspects of the work.

Acknowledgment

Special acknowledgment goes to the Addis Ababa University, College of Health Sciences of the School of Public Health for the support provided to undertake this study. Our deepest gratitude goes to the Addis Ababa city health bureau and selected health institutions.

We also express our gratitude to the staff of all selected health institutions who participated in the study.

References

1. Workbook for Designing, Implementing, and Evaluating a Sharps Injury Prevention Program. CDC; 2008.

2. Gessessew A, Kahsu A. Occupational exposure of health workers to blood and body fluids in six hospitals of Tigray region (August 1-30, 2006): magnitude and management. Ethiopian medical journal. 2009 Jul;47(3):213—219.

3. Dejene H, Kebede F, Mengesha A, Solomon T, Housing and institutional health for environmental health students (Dilla, College of Health Science) November 2002.

4. Nemerow NL, editor. Environmental engineering. Environmental health and safety for municipal infrastructure, land use and planning, and industry. 6th ed. Hoboken, N.J: Wiley; 2009. 556 p.

5. Norsayani MY, Hassim IN. Study on the Incidence of Needle Stick Injury and Factors Associated with this Problem among Medical Students. Journal of Occupational Health. 2003;45(3):172–8.

6. Alonso A. Cementing Sharps Safety in the European Union: The Importance of Complying with the 2010 EU Council Directive on Sharps Injury Prevention. Journal of Nursing & Care. 2014;03(03).

7. Barry S. Levy, David H. Wegman, Rosemary K. Sokas, Heather L. McStowe. Occupational and Environmental Health: Recognizing and Preventing Disease and Injury, seventh edition, Oxford University Press. Oxford University Press; 2017.

8. Lily P, Telu R. Environmental health and hygiene, 2nd edition, Great Britain, 2002; 140-144.

9. International Labor Organization. An ILO Code of Practice on HIV/AIDS and World of Work. Geneva: ILO;p.200.

10. Awases MJ, Nyoni AG, Chatora R. Migration of Health Professionals in Six Countries: A Synthesis Report. Brazzaville: World Health Organization Regional Office for Africa. 2004.

11. Lee JM, Botman MF, Xanthakos N, Nicklasson L. Needlestick injuries in the United States. Epidemiologic, economic, and quality of life issues. AAOHN J. 2005.

12. Cheng H-C, Su C-Y, Yen AM-F, Huang C-F. Cheng HC, Su CY, Yen AM, Huang CF. Factors affecting occupational exposure to needlestick and sharps injuries among dentists in Taiwan: A nationwide survey. PLoS One 2012; 7:e34911. Vermund SH, editor. PLoS ONE. 2012 Apr 3;7(4):e34911.

13. Fishman S, de Onis M, Blössner M, Hyder AA, Hopkins J, Mullany L. Reducing Risks, Promoting Healthy Life. Geneva, Switzerland: WHO; 2012 p. 72–5. :33.

14. Wicker S, Jung J, Allwinn R, Gottschalk R, Rabenau HF. Prevalence and prevention of needlestick injuries among health care workers in a German university hospital. International Archives of Occupational and Environmental Health. 2007 Nov 21;81(3):347–54.

15. Prüss-Üstün A, Rapiti E, Hutin Y. Estimation of the global burden of disease attributable to contaminated sharps injuries among health-care workers. American Journal of Industrial Medicine. 2005 Dec;48(6):482–90.

16. Elizabetta R, Pruss U, Yuvan H. Assessing the burden of disease from sharps injuries to healthcare workers at national and local levels. Geneva: World Health Organization. Environmental Burden of Disease Series. 2005; 11.

17. Tadesse M, Tadesse T. Epidemiology of needlestick injuries among health-care workers in Awassa City, Southern Ethiopia. Tropical Doctor. 2010 Apr;40(2):111–3.

18. Reda AA, Fisseha S, Mengistie B, Vandeweerd J-M. Standard Precautions: Occupational Exposure and Behavior of Health Care Workers in Ethiopia. Tripathy S, editor. PLoS ONE. 2010 Dec 23;5(12):e14420.

19. Yimechew Z, Tiruneh G, Ejigu T. Occupational Exposures to Blood and Body Fluids (BBFS) among Health Care Workers and Medical Students in University of Gondar Hospital, Northwest of Ethiopia. 2013;8.

20. Peng Bi, Tully PJ, Boss K, Hiller JE. Sharps Injury and Body Fluid Exposure Among Health Care Workers in an Australian Tertiary Hospital. Asia Pacific Journal of Public Health. 2008 Apr;20(2):139–47.

21. Goel V, Kumar D, Lingaiah R, Singh S. Occurrence of needlestick and injuries among health-care workers of a tertiary care teaching hospital in North India. Journal of Laboratory Physicians. 2017;9(1):20.

22. Seng M, Sng GKJ, Zhao X, Venkatachalam I, Salmon S, Fisher D. Needlestick injuries at a tertiary teaching hospital in Singapore. Epidemiology and Infection. 2016 Sep;144(12):2546–51.

23. Jahangiri M, Rostamabadi A, Hoboubi N, Tadayon N, Soleimani A. Needle Stick Injuries and their Related Safety Measures among Nurses in a University Hospital, Shiraz, Iran. Safety and Health at Work. 2016 Mar;7(1):72–7.

24. Mbaisi EM, Ng'ang'a Z, Wanzala P, Omolo J. Prevalence and factors associated with percutaneous injuries and splash exposures among health-care workers in a provincial hospital, Kenya, 2010. Pan African Medical Journal. 2013;14.

25. Nwankwo T, Aniebue U. Percutaneous injuries and accidental blood exposure in surgical residents: Awareness and use of prophylaxis about HIV. Nigerian Journal of Clinical Practice. 2011;14(1):34.

26. Share NT, Mulu GB, Habtewold TD, Gizachew KD. Occupational exposure to sharps injury among healthcare providers in Ethiopia regional hospitals. Annals of Occupational and Environmental Medicine. 2017 Dec;29(1).

27. Tadesse M, Merkel M, Boltena AT. Needle-stick and Sharps Injuries among Health Care Workers in Wolaita Zone, Southern Ethiopia. Medical Safety & Global Health. 2016;05(02).

28. Tesafa Bamlie JB. Needle Stick and Sharp Injuries and Associated Factors among Nurses Working In Jimma University Specialized Hospital, South West Ethiopia. Journal of Nursing & Care. 2015;04(05).

29. Kaweti G, Abegaz T. Prevalence of percutaneous injuries and associated factors among health care workers in Hawassa referral and adare District hospitals, Hawassa, Ethiopia, January 2014. BMC Public Health. 2015 Dec;16(1).

30. Bekele T, Gebremariam A, Kaso M, Ahmed K. Attitude, reporting behavior and management practice of occupational needlestick and sharps injuries among hospital healthcare workers in Bale zone, Southeast Ethiopia, Journal of Occupational Medicine and Toxicology. 2015 Dec;10(1).

31. Hospital eTool: Healthcare Wide Hazards - Needlestick/Sharps Injuries [Internet].

32. Motaarefi H, Mahmoudi H, Mohammadi E, Hasanpour-Dehkordi A. Factors Associated with Needlestick Injuries in Health Care Occupations: A Systematic Review. J Clin Diagn Res JCDR. 2016 Aug;10(8): IE01-IE04.

33. Kakizaki M, Ikeda N, Ali M, Enkhtuya B, Tsolmon M, Shibuya K, et al. Needlestick and sharps injuries among healthcare workers at public tertiary hospitals in an urban community in Mongolia. BMC Res Notes. 2011;4(184).

34. Oluwatosin Oa, Oladapo Mm, Asuzu M. Needlestick injuries among health care workers in Ondo State, Nigeria. Int J Med Public Health. 2016;6(1):31.

35 Kelebore WG, Mamo M, Yilma L, Shewalem K, Shakayto S, Kellie M, et al. Assessment of Factors Affecting Needle Stick and Sharp Injuries Among Health Professionals and Cleaners in Arba Minch General Hospital, Gamo Gofa Zone, Southern Ethiopia, 2015 G. C. Sci J Public Health. 2016 Oct 20;4(6):463.

36. Mengesha HB, Yirsaw BD. Occupational Risk Factors Associated with NeedleStick Injury among Healthcare Workers in Hawassa City, Southern Ethiopia. Occup Med Health Aff. 2014 Apr 12;1–5.

37. Walle L, Abebe E, Tsegaye M, Franco H, Birhanu D, Age M. Factors associated with needlestick and sharps injuries, among healthcare workers in Felege Hiwot Referral Hospital, Bahir Dar, Northwest Ethiopia: a facility-based cross-sectional survey. Int J Infect Control. 2013 Oct 17;9(4).

38. Dilie A, Amare D, Gualu T. Occupational Exposure to Needle Stick and Sharp Injuries and Associated Factors among Health Care Workers in Awi Zone, Amhara Regional State, Northwest Ethiopia, 2016. J Environ Public Health; 2017.

39. Bonita R, Beaglehole R, Kjellström T. Basic Epidemiology. 2nd ed. Geneva 27, Switzerland: World Health Organization; 2006.

40. Schoenbach VJ, Rosamond WD. Understanding the Fundamentals of Epidemiology, an evolving text. 2nd ed. Chapel Hill, NC 27599-7400 USA; 2000.

41. Central Statistical Agency Addis Ababa: Ethiopia and ICF International Calverton, Maryland, USA. Ethiopia Demographic and Health Survey; 2016.

42. https://www.ethiopia.gov.etAddis Ababa city profile.

43. Nouetchognou JS, Ateudjieu J, Jemma B, Mbanya D. Accidental exposures to blood and body fluids among health care workers in a Referral Hospital of Cameroon. BMC Research Notes. 2016 Dec;9(1).

44. Alemayehu T, Worku A, Assefa N. Sharp Injury and Exposure to Blood and Body Fluids among Health Care Workers in Health Care Centers of Eastern Ethiopia. The International Journal of Occupational and Environmental Medicine. 2016 Jul 1; 7(3):172–80.

Table 1 - Socio-demographic characteristics of midwives working in government Health institutions of Addis Ababa city, Ethiopia, 2020

Variables	Category	Number	%
Age	20-24	35	8.3
	25-29	219	51.7
	30-34	87	20.5
	35-39	55	12.9
	≥40	28	6.6
Gender	Male	258	60.8
	Female	166	39.2
Marital status	Married	171	40.3
	Single	253	59.7
Educational status	Diploma	14	3.3
	Degree and above	410	96.7
Monthly salary	≤5000	165	38.9
	5001-8000	179	42.2
	≥8001	80	18.9
Year of services	< 5 years	295	69.6
	5-10 years	103	24.3
	≥11	26	6.1

Table 2- Behavior, knowledge, and practice related characteristics of midwives working in public health institutions of Addis Ababa city, Addis Ababa, Ethiopia, 2020

Variables	Categories	Frequency	**%**
Regularly applying universal precautions	Yes	191	45
	No	233	55
Immediate actions following exposure	Washing with soap and water	218	89.3
	Washing with iodine or alcohol solution	29	11.9
	Got tested for HIV	96	39.3
	Took post-exposure prophylaxis (PEP)	42	17.2

Variables	Categories	Frequency	%
	Took tetanus anti-toxoid (TAT)	2	0.8
Personal & patient protection	Wear gloves appropriately	362	85.4
	Proper handwashing	301	71
	Wearing gowns and aprons	300	70.8
	Wearing of eye goggles, masks, and shields	276	65.1
Job-related stress	Yes	192	46
	No	232	54
Job satisfaction	Satisfied	176	42
	Dissatisfied	248	58
Ever had tested for HIV/AIDS	Yes	385	90.8
	No	39	9.2
Ever had tested HBV or HCV	Yes	351	79.8
	No	73	20.2
Appropriate hand wash before putting on gloves	Always	150	35.4
	Sometimes	248	58.5
	Never	26	6.1
Hand wash after handling contaminated objects	Always	294	69.3
	Sometimes	125	29.5
	Never	5	1.2
Hand wash after contact with blood or mucous membrane	Always	347	81.8
	Sometimes	69	16.3
	Never	8	1.9
Availability of antiseptic hand rub	Always	142	33.5
	Sometimes	257	60.6
	Never	25	5.9
Use protective barriers appropriately	Always	191	46.7
	Sometimes	223	52.6

Variables	Categories	Frequency	%
	Never	10	2.4

Table 3 Predictors of BBFs among midwives working in public health institutions of Addis Ababa city, Ethiopia 2020.

Variables	BBFs		COR(95% CI)	AOR(95% CI)
	Yes=198	No=226		
Gender				
Male	163(84.8%)	95(42%)	6.4(4.1-10.1)	2.1(0.99-4.33)
Female	35(15.2%)	131(58%)	1	
Marital status				
Married	50(25.3%)	121(53.5%)	1	
Unmarried	148(74.7%)	105(46.5%)	3.4(2.3-5.2)	6.02(0.42-10.02)
Did you worked in shift?				
Yes	173(87.4%)	173(76.5%)	2.1(1.26-3.6)	2.36(1.034-5.4)*
No	25(12.6)	53(23.5%)	1	1
Work hours per week				
≥40 hours	158(79.8%)	80(35.4%)	7.2(4.6-11.2)	2.01(0.002-9.03)
≤39 hours	40(20.2%)	146(64.6%)	1	
Receiving IP and safety training in the last year				
Yes	27(13.6%)	184(81.4%)	1	1
No	171(86.4%)	42(18.6%)	27.7(16.4-46.9)	4.27(1.94-9.41)***
Use of safety containers regularly				
Yes	16(8.1%)	171(75.6%)	1	1
No	182(91.9%)	55(24.4%)	35.4(19.5-64.1)	21.01(0.99-26.02)
Regularly applying universal precautions				
Yes	11(5.6%)	180(79.6%)	1	1
No	187(94.4%)	46(20.4%)	66.5(33.4-132.5)	8.43(6.55-14.8)***
Job-related stress				
Yes	151(76.3%)	44(19.5%)	13.3(8.4-21.1)	2.18(1.07-4.49)*
No	47(23.7%)	182(80.5%)	1	1
Year of service				
1-5 years	153(77.3%)	142(62.8%)	2.9(1.2-7.2)	0.23(0.25-2.65)

6-10 years	38(19.2%)	65(28.8%)	1.6(0.6-4.1)	
≥ 10 years	7(3.5%)	19(8.4%)	1	1
Monthly salary category				
≤5000 birr	88(44.4%)	77(34%)	1.26(0.74-2.2)	
5001-8000 birr	72(36.4%)	107(47.3%)	0.74(0.437-1.3)	0.305(0.105-0.88)*
≥8001 birr	38(19.2%)	42(18.7%)	1	1
Job satisfaction				
Satisfied	46(23.2%)	132(58.4%)	1	1
Dis satisfied	152(76.8%)	94(41.6%)	4.6(3-7.1)	3.59(0.87-6.07)

**p=0.04; **p=0.01; ***p=0.001*

Click here to access/download
Supporting Information
questionnaire.docx

Occupational ~~Exposure~~exposure to ~~Blood/ Body Fluid Splash~~blood/ body fluid splash and ~~Its Predictors~~its predictors among ~~Midwives Working~~midwives working in ~~Public Health Institutions~~public health institutions at Addis Ababa ~~City~~city Ethiopia, 2020

Institution-based cross-sectional study

[1]Solomon Shitu *, [2]Getachew Adugna, [3]Haimanot Abebe

~~[1, 3]~~[1]Department of Midwifery, Wolkite University College of Health and Medical Sciences, Wolkite University, Wolkite, Ethiopia

[2] City Health Offices, Addis Ababa City ~~Administration~~ Health Departments, Addis Ababa, Ethiopia

[3] Department of Public Health, Wolkite University College of Health and Medical Sciences, Wolkite University, Wolkite, Ethiopia

Email address: [1]solomonsht7@gmail.com,[2]getchadu2013@yahoo.com, [3]haimanotabebe78@gmail.com

Educational qualifications: [1,] Lecturer (BScMw, MSc)

[2, 3] Lecturer (BSc public health, Mph)

*Corresponding Author

Corresponding Author Address

Email address: solomonsht7@gmail.com

Cell phone +251924704053

Postal address 07 Wolkite, Ethiopia

Living address wolkite town

Formatted: Header

Formatted: Font: 18 pt

Formatted: Font: 18 pt

Formatted: Font: 18 pt

Formatted: Font: 18 pt

Formatted: Font: 18 pt

Formatted: Font: 18 pt

Formatted: Font: 18 pt

Formatted: Not Superscript/ Subscript

Formatted: Header

Formatted: Font: +Body (Calibri), 11 pt, Not Bold

Formatted: Normal

Abstract

Formatted: Font: 18 pt

Formatted: Font: 14 pt

Background: Blood/body fluid splash are hazards to health care professionals in their working area. Around twenty bloodborne pathogens are known to be transmitted through these occupational injuries. This problem alters the health status of health care professionals in different ways, including physically, mentally, and psychologically. Even though health ~~care~~ professionals especially midwives who are working in delivery rooms are highly affected~~ at a high rate, there~~, little is ~~a low injury report to a higher level.~~known about the exposure. So, this study was aimed to assess the prevalence of ~~occupational~~ exposure to blood/body fluid splash and its predictors among midwives working in public health institutions of Addis Ababa city.

Formatted: Heading 2 Char, Font: 16 pt, Not Bold

Methods: Institution based cross-sectional study was conducted among 438 study participants in public health institutions in Addis Ababa. Data was collected from March 1-20, 2020 by a self-administered questionnaire. The data were entered into Epi data version 3.1 and then exported to SPSS version 24 for analysis. All variables with $P<0.25$ in the bivariate analysis were included in a final model and statistical significance was declared at $P< 0.05$.

Formatted: Heading 2 Char, Font: 16 pt, Not Bold

Results: In this study, a total of 424 respondents respond yielding a response rate of 97%. The prevalence of blood and body fluid splashes (BBFs) was 198 (46.7%). Not training on infection prevention, working in two~~ shift,~~ shifts (> 12 hours), not regularly

Formatted: Heading 2 Char, Font: 16 pt, Not Bold

apply universal precautions, job-related stress, an average monthly salary of 5001-8000 were independent predictors of blood and body fluid splashes.

Conclusion: The burdenstudy revealed that nearly half of blood/body fluid splash was high (46.7%). Not training on infection prevention, working in two shift, not regularly apply universal precautions, job-related stress, an average monthly salary of 5001-8000midwives were independent predictors. Unsafe practiceexposed to BBFS. This sparks light to concerned bodies like policymakers and not applying universal precautions were also considerably high.stack holders to design appropriate policy to avert this magnitude and making the environment enabling to comply with standard precautions. We recommend that this study may be done by including rural setting institutions and by including other health professionals that are susceptible to BBFS at work. Formal training on infection prevention and safety practice to health care professionals apply universal precautions will be needed from the concerned bodies is mandatory to prevent exposures to blood/body fluid splash.

Keywords: Blood/body fluid splash, midwives, Addis Ababa

Formatted: Header

Formatted: Heading 2 Char, Font: 16 pt, Not Bold

Formatted: Heading 2 Char, Font: 16 pt, Not Bold

Background

Introduction

Blood/body fluid splash (BBFS) are hazards to health care workers in their working area. Around twenty bloodborne pathogens are known to transmit through thesethis occupational injuriesexposure [1]. This problem alters the health status of health care professionals (HCPs) in different ways, including physically, mentally, and psychologically. Even though HCPs are affected at a high rate, there is a low injury report to a higher level [2].

No occupation or work is free from health hazards. People are always under a certain amount of health risk wherever they are working [3]. Health Care Professionalscare professionals are exposed to a wide range of occupational health and safety hazards. [4]. An estimated 20 million work-related injuries and 390, 000 new work-related illnesses occur each year in the United States and China 66.3% of HCPs per year exposed to BBFS; however, the number of occupational diseases and injuries reported each year is much more [7]. 4, 5]. No occupation or work is free from health hazards. People are always under a certain amount of health risk wherever they may be working [8].

Nowadays, workplace injuries, especially blood and body fluidsBBFS prevalence, isare increasing among health workers [6, 7]. Occupational exposure to blood/body fluidsBBFS in healthcare facilities constitutes a significant risk of transmission of human immune deficiency virus (HIV) and other blood-borne pathogens to healthcare workers [9, 8-10]

As estimated by the Center for Disease Control (CDC) that every year more than three million Health Care providersHCPs are openedexposed to blood and body fluidsBBFs, through sharps, plus Mucocutaneousmucocutaneous injuries in the United States [10]. Due to BBFsBBFS, the possibility of infections ranges from as low as 0.2–0.5% for HIV to as huge as 3–10% for Hepatitis C virus (HCV) and 40% for HBV(11). The quantity of HCWs annually exposed to sharps injuries contaminated with hepatitisHepatitis B virus, hepatitis C virus, and human immunodeficiency virus/AIDS (HBV) [11]. World health organization (WHO) estimates that nearly 3 million HCPs experience occupational exposure to blood-borne viruses HBV, HCV, and HIV has been reported to be 2.1 million, 926,000, and 327,000 [14, respectively [12-15]. From

Formatted: Header

Formatted: Normal, Space Before: 1 line, After: 1 line

Formatted: Header

this 90% of this exposure to infection was resulted from low resource countries [14]. In Ethiopia, studies indicated that the prevalence of HCV due to BBFS exposure was 0.9- 5.8% [3, 16].

Health care workers in Africa ~~suffer~~experience from two to four needle-stick injuries per year on average, with Nigeria, Tanzania, and South Africa reporting on average of 2.10 injuries per ~~HCW on average (16).~~
~~Previous studies in Eastern, Southern, and Northern parts of Ethiopia have shown the increased risk of occupational blood exposure~~ HCPs per year [17~~-19].~~].
~~In Ethiopia,~~Even though there is no ~~represented~~ national data regarding the prevalence of ~~BBFs among health care professionals.~~BBFS among midwives in Ethiopia previous studies in the Eastern part of the country showed that the prevalence of BBFS was 28.8% [18]. The majority of ~~the~~these studies were only confined to a single institution and ~~determinants contributing to~~ workplace ~~injuries have been given little attention~~ [18, 19]. A high rate of midwives is affected by the exposure [2]. Despite the presence of many possibilities to reduce ~~disease~~ the burden~~, the full consequence~~ of occupational exposure to ~~blood/body fluid splash~~BBFS, it is not yet recognized. Hence this research was carried out ~~to determine the prevalence and determinants of BBFs, among~~on midwives working ~~in~~at public health institutions of Addis Ababa city due to they are more exposed and the first line for BBFS.

Formatted: Expanded by 0.05 pt

Formatted: Condensed by 0.05 pt

Methods

Formatted: Font: 18 pt

Study ~~Design, Area~~design, area, and ~~Period~~period

Formatted: Font: 16 pt

Formatted: Font: 16 pt

Formatted: Font: 16 pt

An institution-based cross-sectional study was conducted from March 1-20, 2020 in selected public health institutions of Addis Ababa ~~city. Addis Ababa is the~~Ethiopia's capital city~~ of the country of Ethiopia.~~. It comprises 10 sub-cities. In the City, there are 12 public hospitals and 112 health centers. All institutions serve the population of Addis Ababa and provide all services including maternal and child health care service [42].

Population

Formatted: Font: 16 pt

Source ~~Population~~population

Formatted: Font: 14 pt

Formatted: Font: 14 pt

All midwives working in public health institutions of Addis Ababa city.

Study ~~Population~~population

Randomly selected midwives working in public health institutions of Addis Ababa city.

Inclusion ~~Criteria~~criteria

All ~~licensed /registered~~ midwives working in Addis Ababa city

Exclusion ~~Criteria~~criteria

Midwives who were on annual or maternity leave and who were critically ill and unable to respond during data collection.

Sample size determination

~~A separate~~Separate sample size was calculated for each specific objective by using both single and double population proportion formula ~~by using epi info 7.1 stat cal.~~ was used. For the first specific objective, (To determine the prevalence of occupational exposure to BBFS among midwives working in Addis Ababa city) single population proportion was used.

$n = \frac{(Z\,\alpha/2)^2\, P\,(1-P)}{d^2}$ *by the* assumption of n= minimum sample size required for the study

$(Z\ \alpha/2)^2$ = standard normal distribution with 95% confidence interval, P= 21% (prevalence of occupational exposure and behavior of health care workers in Ethiopia) [18], d= is a tolerable margin of error (d=0.04)

The sample size of the first objective was greater than that of the second objective. By adding a 10% non-response rate from the largest sample size the calculated sample size was 438.

Sampling procedure

Six public hospitals from 12 hospitals and 66 health centers from 112 health centers were selected by simple random sampling technique. The total number of midwives working in Addis Ababa city public health institutions was 2432 and the number of midwives working in selected public health institutions was 1296. To select study participants from selected health institutions the proportional allocation method (each institution's number of midwives was allocated by proportion) was used for each ~~public health~~ institution. Study participants who fulfilled the

Formatted: Header
Formatted: Font: 14 pt
Formatted: Font: 14 pt
Formatted: Font: 16 pt
Formatted: Heading 2
Formatted: Font: Times New Roman, 16 pt, Bold, Italic
Formatted: Font: Bold, Italic
Formatted: Font: 16 pt
Formatted: Font: 16 pt
Formatted: Font: 16 pt, Bold, Italic
Formatted: Font: 16 pt
Formatted: Font: 16 pt

Formatted: Header

inclusion criteria were selected by simplesystematic random sampling technique until the required number is obtained. with a kth value of 3. The registers of all midwives were obtained from the administration of the offices. Any unwilling or unavailable selected participant was replaced by the very next person on the list who had not been already selected to participate.

Dependent variables

Formatted: Font: 16 pt
Formatted: Font: 16 pt, Bold
Formatted: Heading 2, Space Before: 0 pt

Exposure to blood/body fluid splash

Independent variables

Formatted: Font: 16 pt
Formatted: Heading 2, Space Before: 0 pt
Formatted: Font: 16 pt, Bold

Socio-demographic factors: (Age, Sex, Marital status, Qualifications of midwives, Monthly income)

Work-related factors: (Training on IP, work areaworking environment, year of service, educational level)

Behavioral factors: (Job satisfaction, reporting after the exposure, using the universal precautions and personal protective methodequipment)

Data quality control and collection methods

Formatted: Font: 16 pt

The questionnaire was developed by reviewing different pieces of literature. [2, 3, 7, 17, 18]. Data were collected by facilitator guided structured self-administered English version questionnaire. Facilitators took two days of training to assure quality. The questionnaire was checked for completeness before data entry into the software. Proper coding and categorization of data were maintained for the quality of the data to be analyzed. Double data entry was done for its validity and compare them to the original data.

Data processing and analysis

Formatted: Font: 16 pt

The collected data were checked and reviewed for completeness, coded, and entered into Epi data version 3.1, and exported to SPSS version 24 for analysis. Descriptive statistics were used to determine the frequency of different variables. Association between dependent and independent variables were examined using bi-variable and multivariable logistic regression models. Those variables which were (P-value < 0.25) on bi-variate[18] in the bivariate analysis were entered

into multivariable logistic regression analysis. included in the final model of multivariate analysis to control all possible confounders. Multi-collinearity was checked to see the linear correlation among the independent variables by using standard error. Variables with a standard error of > 2 were dropped from the multivariable analysis. Model fitness was checked with the Hosmer-Lemeshow test. The direction and strength of statistical association were measured by the odds ratio with 95 % CI. using multivariable logistic regression analysis. Adjusted odds ratio along with 95% CI was estimated to identify factors for BBFs by using multivariate analysis in the binary logistic regression.associated with BBFS. In this study P-value < 0.05 was considered to declare a result as statistically significant.

Operational Definitions

Blood/Body exposure: those exposed to blood, vomits, urine, sputum, saliva, amniotic fluid, exudative fluids ([31).].

Personal protective equipment: barriers and filters between the worker and the hazard ([1).].

Ethical consideration and consent to participate

Ethical clearances were obtained from institutional review boards (IRB) of Addis Ababa University. An official letter of permission was submitted to the Addis Ababa city health bureau. A formal letter was submitted to selected public health institutions from the bureau. The respondents were informed about the objective, purpose, risks, and benefits of the study and the right to refuse to participate, and then informed written and signed consent was taken.

The study posed a low or no more than minimal risk to the study participants. Also, the study did not involve any invasive procedures. Moreover, the confidentiality of information was guaranteed by using code numbers rather than personal identifiers and by keeping the data locked.

Formatted: Header

Formatted: Font: 16 pt

Formatted: Font: 16 pt

Formatted: Normal, Space Before: 1 line, After: 1 line

Results

Socio-demographic characteristics

A total of 424 respondents ~~have filled~~completed the questionnaire yielding a response rate of 97%. The age distribution of the respondents ranges from 20-56 years, with a mean and median age of 30.11 ~~years~~ (SD ± 5.37~~)~~), 26.5 years respectively, and 219 (51.7~~%).~~%) were between the age group of 25-29 years. Nearly two-thirds of participants, 258 (60.8%) were males. Unmarried accounted for 253(59.7%). In this study majority, four hundred and ten (96.7%) participants were holders of degrees and above. More than four out of five earned a monthly salary of ≤8000 Ethiopian birrs. Two-third, 295 (69.6%), had less than five years' work experience (Table 1).

Behavior, knowledge, and practice of midwives

Out of the respondents, less than half, 191(45%) responded that they had followed universal precautions regularly, and 187 (44.1%) usually use safety ~~box.~~deposit boxes to discard sharp materials. Concerning the protection of oneself and the patient from infection, more than three fourth 362 (85.4%) of respondents indicated that they wear gloves appropriately. The wearing of gowns and aprons had responded by 300(70.8%). Among study participants, 195 (46~~%~~%) had responded that they had job-related stress, and 246 (58%) answered that they were dissatisfied with their job. More than two-thirds of 294 (69.3%) of participants reported that they had always washed their hands immediately after handling contaminated objects while 246 (58.5~~%~~%) always wash their hands before putting on gloves. Three hundred forty-seven 347 (81.8%) reported that they washed their hands always after contact with blood or mucous membrane (Table 2).

Prevalence of blood/body fluid splashes

From a total of 424 midwives who participated in the study occupational exposure to blood/body fluid splash at least once was 198 (46.7%) with 95%CI: 41.3-52.2. Among those exposed to ~~BBFs~~BBFS in the last ~~one~~ year, 163(82.3%) of them encountered one to two times, and 14 (7.1%) faced five and more times. The status of the source of exposure was unknown for 79 (39.5%), whereas, 25(12.5%) were known HIV positive, 16 (8%) were clinically suspected HIV positive, however, 77(38.5%) known HIV negative. Only, 3 (1.5%) of the source of exposure

Formatted: Header

Formatted: Font: 18 pt

Formatted: Font: 16 pt

Formatted: Space After: 1 pt

Formatted: Font: 11 pt

Formatted: Font: 16 pt

Formatted: Font: 16 pt, Font color: Auto

Formatted: Heading 2, None, Space Before: 0 pt, Don't keep with next, Don't keep lines together

Formatted: Font: 16 pt, Not Bold

was reported unknown hepatitis B positive. Of those BBFS, 138 (60.8%) exposure was encountered to skin. The splash to the broken membrane of the skin, eyes, and mouth were 16 (7.9%), 51 (25.6%), 11 (5.7%) respectively.

Among those exposed to BBFs, 163(82.3%) of them encountered one to two times, and 14 (7.1%) faced five and more times. Of the total BBFs exposed, the status of the source of exposure 79(39.5%) was unknown, whereas, 25(12.5%) were known HIV/AIDS positive, 16(8%) were clinically suspected HIV/AIDS positive, however, 77(38.5%) known HIV/AIDS negative. Only, 3(1.5%) reported having hepatitis B positive status. Of those BBFs, 138(60.8%) exposure was encountered to skin. The splash to the broken membrane of skin (7.9%), eyes (25.6%), and to face and mouth (5.7%) were reported

Predictors of **Blood/body fluid splash**

Formatted: Header

Formatted: Font: 16 pt

According to bivariate regression analysis being male, single marital status, working in shifts, worked ≥40 hours per week, presence of IP committee in hospital, not trained for IP and safety in last 1 year, not use of safety containers regularly, not applying universal precaution, presence of job-related stress, year of service, monthly salary and job dissatisfaction were statistically associated with blood/body fluid splash with p-value ≤0.05. In

Formatted: Font: 16 pt, Font color: Auto

According to multivariable logistic regression found that working in two shifts,(working for > 12 hours), not receive infection preventionreceived IP and safety practice training, not regularly apply universal precautions, job-related stress, an average monthly salary of 5001-8000 were independent predictors of BBFsBBFS.

The odds ratio was twoHealth care professionals who did not regularly apply universal precautions were 8 times higher for those working in shiftmore likely to have blood/body fluid splash (AOR 2.368, 95% CI 1.034-5.46.55 - 14.8) than nottheir counterparts. Participants who did not receive IP and safety practice training were four times more likely to have blood/body fluid splash (AOR 4.27, 95% CI 1.94 - 9.41) than those who did receive training. Health care professionals who did not regularly apply universal precautions were 8The odds ratio was two times higher for those working in two shifts (more likely to have blood/body fluid splashthan 12

Formatted: Header

hours) (AOR ~~8~~2.36, 95% CI ~~6.55 - 14.8~~1.034-5.4) than ~~their counterparts.~~ one shift (≤ 12 hours). The odds of exposure to BBFs were two times increased for those who had job-related stress (AOR 2.18, 95% CI 1.07-4.49) than their counterpart. Respondents who get an average monthly salary of 5001-8000 Ethiopian birr were 70% less likely to be exposed to ~~blood/body fluid splash~~BBFS (AOR 0.305, 95% CI 0.105 - 0.88) than those who had gained less than 8000 Ethiopian birrs~~. Gender, marital status, working in a shift, presence of infection prevention committee in hospital, use of safety containers regularly, year of service, and job satisfaction were not statistically significant~~ (Table 3).

Discussions

Formatted: Font: 18 pt

In this study, findings indicate that the prevalence of ~~BBFs~~BBFS was 198 (46.7%) ~~HCPs reported having been~~with 95%CI: 41.3-52.2 exposed ~~to blood and body fluid splashes~~ at least once~~.~~ in their work life. The prevalence was in line with the study done in Australia 42% [20]. But it was significantly higher than some findings in Eastern Ethiopia (20.2% and elsewhere 36.1%) [~~17~~18, 44]. In contrast, this proportion remains low compared to the study done in Gonder and Tigray Ethiopia at 62.9%, 56.3% respectively [2, 19]. The reason for this difference could be methodological differences and variation in study area and periods, and under-reporting in this study. And also may be due to the difference in ~~work cultures and the availability of resources.~~and the availability of resources. Thus, the study was done in an urban area the availability of materials to apply safety precautions was better than in rural settings. Work cultures may be significantly affecting the outcome the study included only midwives in whose work area is more susceptible for BBFS.

The most potential determinant factor associated with BBFS was not regularly applying universal precaution (AOR 8.43, 95% CI 6.55-14.8). Those who did not apply universal precautions were 8 times more exposed to BBFS than those who apply. This is consistent with studies done in Mongolia [33] and Northwest Ethiopia [38]. This might be because those who did not adhere to universal precautions were more likely to incur occupational exposure due to they are working unprotected.

In this study, midwives who worked in two shifts (≥12 hours) were twice more likely to be exposed to ~~BBFs~~BBFS (AOR 2.36, 95% CI 1.034-5.4) than those who work in ~~three shifts.~~only

one shift (≤12 hours). This finding was supported by the study done in Germany [14], and Ondo state, Nigeria [34]. Since two-shift work has a high clinical activity schedule, and with full of workload and they will stay more than 12 hours in the workplace, so as a result, professionalstime elapses they become tired and negligent to useapply universal precautions.

The result of the study indicates that lack of training on IP and safety practice in the last year was a significant determinant to BBFsBBFS (AOR 4.27, 95% CI 1.94-9.41). This may be because training enhances or contributed to change the knowledge attitude and practice of individuals. Similarly, a study carried out in Debre Birhan Ethiopia and Kenya [24, 26] revealed that the absence of onsite training on IP and safety practice was the main contributor to occupational exposure. Also maybe knowledge and practice acquired during training necessarily translate into practice on prevention measures and safety precautions.

The most potential determinant associated with BBFs was not regularly applying universal precaution (AOR 8.43, 95% CI 6.55-14.8). This is consistent with studies done in Mongolia [33] and Northwest Ethiopia [38]. This may be due to those who did not adhere to universal precautions were more likely to incur occupational exposure.

Midwives who had job-related stress were two times more likely to be exposed to BBFsBBFS (AOR 2.18, 95% CI 1.07-4.49) than those who did not have. job-related stress. This is in agreement with the study done in Northwest Ethiopia [38]. This may be justified since those who had job-related stress could not focus on their jobs and they may be predisposed to BBFsBBFS. And also stress may lead to a lack of concentration on work.

Respondents who earned a monthly salary of 5000-8000 birr were 70% less likely to be exposed to BBFsBBFS (AOR 0.30, 95% CI 0.105-0.88) when compared to respondents who got a monthly salary of ≤5000. Possibly may be due to their satisfaction with their monthly salary, they might be involved seriously in their activities. Also, those who earned ≤5000 might be those with newly deployed to work so they may be less experienced and training could be given first for those senior staffs.

Formatted: Header

Formatted: Tab stops: 2.74", Left

Conclusion

This The study aimed to assess the burdenrevealed that nearly half of occupational exposure and associated determinants to blood/body fluid splash. The prevalence of blood/body fluid splash was high 46.7%. Not training on infection prevention, working in two shift, not regularly apply universal precautions, job related stress, an average monthly salary of 5001-8000midwives were independent predictors. Unsafe practiceexposed to BBFS. This sparks light to concerned bodies like policymakers and stack holders to design appropriate policy to avert this magnitude and not applying universal precautions were also considerably high.making the environment enabling to comply with standard precautions. Also, this study was done in urban health settings, and the institutions are better equipped with personal protective materials than the rural settings. So, we recommend that this study may be done by including rural setting institutions and by including other health professionals that are susceptible to BBFS at work. Formal training on infection prevention and safety practice to health care professionals apply universal precautions will be needed from the concerned bodies is mandatory to prevent exposures to blood/body fluid splash.

Strength and limitation of the study

Strength of study

This study provides information about the burden of occupational exposure related to BBFs. It is an unsearched/ neglected thematic area of the health care system and it adds value for the system and serves as a baseline for researchers.

Limitation of study

Information was obtained by asking the respondents to recall whether they had faced it or not,, so this may incur recall bias. And due to the self-administered questionnaire, it was susceptible to social desirability bias HCPs might report socially acceptable responses than their actual day-to-day practice that may underestimate the prevalence of the outcome.

The study was included only urban setting institutions and midwives working there so it lacks generalizability including midwives working in the rural area. And the study has done only on midwives so better to include other health professionals.

Formatted: Header

Formatted: Font: 18 pt

Formatted: Heading 1

Formatted: Font: Helvetica, 10 pt, Font color: Gray-87.5%, Pattern: Clear (White)

Formatted: Font: 18 pt

Formatted: Font: 16 pt

Formatted: Font: 16 pt

Formatted: Header

Because of the cross-sectional study design, the study could not answer the direction of causality between independent factors and outcome.

~~Acronyms and~~ Abbreviations

Formatted: Font: 18 pt

AIDS	Acquired Immune Deficiency Syndrome
AOR	Adjusted Odds Ratio
~~BBFs~~BBFS	Blood/Body Fluid splash
CDC	Center for Disease Control
CI	Confidence Interval
COR	Crude Odds Ratio
~~EPINet~~	~~Exposure Prevention Information Network~~
FMoH	Federal Ministry of Health
HBV	Hepatitis B virus
HCV	Hepatitis C virus
~~HCWs~~HCPs	Health Care ~~Workers~~Providers
HIV	Human Immunodeficiency Virus
IP	Infection Prevention
OR	Odds Ratio
PEP	Post Exposure Prophylaxis
SPSS	Statistical Package for the Social Sciences
WHO	World Health Organization

Declarations

The content of the study is solely the responsibility of the authors.

~~Ethical consideration and consent to participate~~

~~Ethical clearances were obtained from institutional review boards (IRB) of Addis Ababa University. An official letter of permission was submitted to the Addis Ababa city health bureau. A formal letter was submitted to selected public health institutions from the bureau. The respondents were informed about the objective, purpose, risks, and benefits of the study and the right to refuse to participate, and then informed written and signed consent was taken.~~

~~The study posed a low or no more than minimal risk to the study participants. Also, the study did not involve any invasive procedures. Moreover, the confidentiality of information was guaranteed by using code numbers rather than personal identifiers and by keeping the data locked.~~

Consent for publication

Not applicable

Availability of data and materials

The datasets used and/or analyzed during the current study are available from the corresponding author on reasonable request.

Competing interests

The authors would like to declare that we have no competing interest in this study.

Funding

There is no source of funding for this research. All costs were covered by researchers.

Formatted: Header

Formatted: Font: 18 pt, Not Bold, Font color: Auto

Formatted: Font: Times New Roman, 18 pt

Formatted: Heading 1, Adjust space between Latin and Asian text, Adjust space between Asian text and numbers

Formatted: Font: 16 pt

Formatted: Normal, Space Before: 1 line, After: 1 line

Formatted: Font: 18 pt

Formatted: Font: 18 pt, Not Bold, Font color: Auto

Formatted: Heading 1, Line spacing: single, Adjust space between Latin and Asian text, Adjust space between Asian text and numbers

Formatted: Font: 18 pt, Not Bold

Formatted: Font: 18 pt, Not Bold, Font color: Auto

Formatted: Heading 1, Line spacing: single, Adjust space between Latin and Asian text, Adjust space between Asian text and numbers

Formatted: Font: 18 pt, Not Bold

Formatted: Font: 18 pt, Not Bold, Font color: Auto

Formatted: Font: 18 pt, Not Bold

Formatted: Heading 1, Line spacing: single, Adjust space between Latin and Asian text, Adjust space between Asian text and numbers

Formatted: Header

Authors' contributions

Formatted: Font: 18 pt, Not Bold, Font color: Auto

Formatted: Font: 18 pt, Not Bold

Formatted: Heading 1, Line spacing: single, Adjust space between Latin and Asian text, Adjust space between Asian text and numbers

All authors made a significant contribution to the work reported, whether that is in the conception, study design, execution, acquisition of data, analysis, and interpretation, or in all these areas; took part in drafting, revising, or critically reviewing the article; gave final approval of the version to be published; have agreed on the journal to which the article has been submitted; and agree to be accountable for all aspects of the work.

Acknowledgment

Formatted: Font: 18 pt

Special acknowledgment goes to the Addis Ababa University, College of Health Sciences of the School of Public Health for the support provided to undertake this study. Our deepest gratitude goes to the Addis Ababa city health bureau and selected health institutions.

We also express our gratitude to the staff of all selected health institutions who participated in the study.

References

Formatted: Font: 18 pt

1. Workbook for Designing, Implementing, and Evaluating a Sharps Injury Prevention Program. CDC; 2008.

2. Gessessew A, Kahsu A. Occupational exposure of health workers to blood and body fluids in six hospitals of Tigray region (August 1-30, 2006): magnitude and management. Ethiopian medical journal. 2009 Jul;47(3):213—219.

3. Dejene H, Kebede F, Mengesha A, Solomon T, Housing and institutional health for environmental health students (Dilla, College of Health Science) November 2002.

4. Nemerow NL, editor. Environmental engineering. Environmental health and safety for municipal infrastructure, land use and planning, and industry. 6th ed. Hoboken, N.J: Wiley; 2009. 556 p.

5. Norsayani MY, Hassim IN. Study on the Incidence of Needle Stick Injury and Factors Associated with this Problem among Medical Students. Journal of Occupational Health. 2003;45(3):172–8.

6. Alonso A. Cementing Sharps Safety in the European Union: The Importance of Complying with the 2010 EU Council Directive on Sharps Injury Prevention. Journal of Nursing & Care. 2014;03(03).

7. Barry S. Levy, David H. Wegman, Rosemary K. Sokas, Heather L. McStowe. Occupational and Environmental Health: Recognizing and Preventing Disease and Injury, seventh edition, Oxford University Press. Oxford University Press; 2017.

8. Lily P, Telu R. Environmental health and hygiene, 2nd edition, Great Britain, 2002; 140-144.

9. International Labor Organization. An ILO Code of Practice on HIV/AIDS and World of Work. Geneva: ILO;p.200.

10. Awases MJ, Nyoni AG, Chatora R. Migration of Health Professionals in Six Countries: A Synthesis Report. Brazzaville: World Health Organization Regional Office for Africa. 2004.

11. Lee JM, Botman MF, Xanthakos N, Nicklasson L. Needlestick injuries in the United States. Epidemiologic, economic, and quality of life issues. AAOHN J. 2005.

12. Cheng H-C, Su C-Y, Yen AM-F, Huang C-F. Cheng HC, Su CY, Yen AM, Huang CF. Factors affecting occupational exposure to needlestick and sharps injuries among dentists in Taiwan: A nationwide survey. PLoS One 2012; 7:e34911. Vermund SH, editor. PLoS ONE. 2012 Apr 3;7(4):e34911.

13. Fishman S, de Onis M, Blössner M, Hyder AA, Hopkins J, Mullany L. Reducing Risks, Promoting Healthy Life. Geneva, Switzerland: WHO; 2012 p. 72–5. :33.

14. Wicker S, Jung J, Allwinn R, Gottschalk R, Rabenau HF. Prevalence and prevention of needlestick injuries among health care workers in a German university hospital. International Archives of Occupational and Environmental Health. 2007 Nov 21;81(3):347–54.

15. Prüss-Üstün A, Rapiti E, Hutin Y. Estimation of the global burden of disease attributable to contaminated sharps injuries among health-care workers. American Journal of Industrial Medicine. 2005 Dec;48(6):482–90.

Formatted: Header

16. Elizabetta R, Pruss U, Yuvan H. Assessing the burden of disease from sharps injuries to healthcare workers at national and local levels. Geneva: World Health Organization. Environmental Burden of Disease Series. 2005; 11.

17. 17. Tadesse M, Tadesse T. Epidemiology of needlestick injuries among health-care workers in Awassa City, Southern Ethiopia. Tropical Doctor. 2010 Apr;40(2):111–3.

18. Reda AA, Fisseha S, Mengistie B, Vandeweerd J-M. Standard Precautions: Occupational Exposure and Behavior of Health Care Workers in Ethiopia. Tripathy S, editor. PLoS ONE. 2010 Dec 23;5(12):e14420.

18. Tadesse M, Tadesse T. Epidemiology of needlestick injuries among health-care workers in Awassa City, Southern Ethiopia. Tropical Doctor. 2010 Apr;40(2):111–3.

19. Yimechew Z, Tiruneh G, Ejigu T. Occupational Exposures to Blood and Body Fluids (BBFS) among Health Care Workers and Medical Students in University of Gondar Hospital, Northwest of Ethiopia. 2013;8.

20. Peng Bi, Tully PJ, Boss K, Hiller JE. Sharps Injury and Body Fluid Exposure Among Health Care Workers in an Australian Tertiary Hospital. Asia Pacific Journal of Public Health. 2008 Apr;20(2):139–47.

21. Goel V, Kumar D, Lingaiah R, Singh S. Occurrence of needlestick and injuries among health-care workers of a tertiary care teaching hospital in North India. Journal of Laboratory Physicians. 2017;9(1):20.

22. Seng M, Sng GKJ, Zhao X, Venkatachalam I, Salmon S, Fisher D. Needlestick injuries at a tertiary teaching hospital in Singapore. Epidemiology and Infection. 2016 Sep;144(12):2546–51.

23. Jahangiri M, Rostamabadi A, Hoboubi N, Tadayon N, Soleimani A. Needle Stick Injuries and their Related Safety Measures among Nurses in a University Hospital, Shiraz, Iran. Safety and Health at Work. 2016 Mar;7(1):72–7.

24. Mbaisi EM, Ng'ang'a Z, Wanzala P, Omolo J. Prevalence and factors associated with percutaneous injuries and splash exposures among health-care workers in a provincial hospital, Kenya, 2010. Pan African Medical Journal. 2013;14.

Formatted: Header

Formatted: Header

25. Nwankwo T, Aniebue U. Percutaneous injuries and accidental blood exposure in surgical residents: Awareness and use of prophylaxis about HIV. Nigerian Journal of Clinical Practice. 2011;14(1):34.

26. Share NT, Mulu GB, Habtewold TD, Gizachew KD. Occupational exposure to sharps injury among healthcare providers in Ethiopia regional hospitals. Annals of Occupational and Environmental Medicine. 2017 Dec;29(1).

27. Tadesse M, Merkel M, Boltena AT. Needle-stick and Sharps Injuries among Health Care Workers in Wolaita Zone, Southern Ethiopia. Medical Safety & Global Health. 2016;05(02).

28. Tesafa Bamlie JB. Needle Stick and Sharp Injuries and Associated Factors among Nurses Working In Jimma University Specialized Hospital, South West Ethiopia. Journal of Nursing & Care. 2015;04(05).

29. Kaweti G, Abegaz T. Prevalence of percutaneous injuries and associated factors among health care workers in Hawassa referral and adare District hospitals, Hawassa, Ethiopia, January 2014. BMC Public Health. 2015 Dec;16(1).

30. Bekele T, Gebremariam A, Kaso M, Ahmed K. Attitude, reporting behavior and management practice of occupational needlestick and sharps injuries among hospital healthcare workers in Bale zone, Southeast Ethiopia, Journal of Occupational Medicine and Toxicology. 2015 Dec;10(1).

31. Hospital eTool: Healthcare Wide Hazards - Needlestick/Sharps Injuries [Internet].

32. Motaarefi H, Mahmoudi H, Mohammadi E, Hasanpour-Dehkordi A. Factors Associated with Needlestick Injuries in Health Care Occupations: A Systematic Review. J Clin Diagn Res JCDR. 2016 Aug;10(8): IE01-IE04.

33. Kakizaki M, Ikeda N, Ali M, Enkhtuya B, Tsolmon M, Shibuya K, et al. Needlestick and sharps injuries among healthcare workers at public tertiary hospitals in an urban community in Mongolia. BMC Res Notes. 2011;4(184).

34. Oluwatosin Oa, Oladapo Mm, Asuzu M. Needlestick injuries among health care workers in Ondo State, Nigeria. Int J Med Public Health. 2016;6(1):31.

Formatted: Header

35 Kelebore WG, Mamo M, Yilma L, Shewalem K, Shakayto S, Kellie M, et al. Assessment of Factors Affecting Needle Stick and Sharp Injuries Among Health Professionals and Cleaners in Arba Minch General Hospital, Gamo Gofa Zone, Southern Ethiopia, 2015 G. C. Sci J Public Health. 2016 Oct 20;4(6):463.

36. Mengesha HB, Yirsaw BD. Occupational Risk Factors Associated with NeedleStick Injury among Healthcare Workers in Hawassa City, Southern Ethiopia. Occup Med Health Aff. 2014 Apr 12;1–5.

37. Walle L, Abebe E, Tsegaye M, Franco H, Birhanu D, Age M. Factors associated with needlestick and sharps injuries, among healthcare workers in Felege Hiwot Referral Hospital, Bahir Dar, Northwest Ethiopia: a facility-based cross-sectional survey. Int J Infect Control. 2013 Oct 17;9(4).

38. Dilie A, Amare D, Gualu T. Occupational Exposure to Needle Stick and Sharp Injuries and Associated Factors among Health Care Workers in Awi Zone, Amhara Regional State, Northwest Ethiopia, 2016. J Environ Public Health; 2017.

39. Bonita R, Beaglehole R, Kjellström T. Basic Epidemiology. 2nd ed. Geneva 27, Switzerland: World Health Organization; 2006.

40. Schoenbach VJ, Rosamond WD. Understanding the Fundamentals of Epidemiology, an evolving text. 2nd ed. Chapel Hill, NC 27599-7400 USA; 2000.

41. Central Statistical Agency Addis Ababa: Ethiopia and ICF International Calverton, Maryland, USA. Ethiopia Demographic and Health Survey; 2016.

42. https://www.ethiopia.gov.etAddis Ababa city profile.

43. Nouetchognou JS, Ateudjieu J, Jemma B, Mbanya D. Accidental exposures to blood and body fluids among health care workers in a Referral Hospital of Cameroon. BMC Research Notes. 2016 Dec;9(1).

44. Alemayehu T, Worku A, Assefa N. Sharp Injury and Exposure to Blood and Body Fluids among Health Care Workers in Health Care Centers of Eastern Ethiopia. The International Journal of Occupational and Environmental Medicine. 2016 Jul 1; 7(3):172–80.

Formatted: Header

Table 1 - Socio-demographic characteristics of midwives working in government Health institutions of Addis Ababa city, Ethiopia, 2020

Variables	Category	Number	%
Age	20-24	35	8.3
	25-29	219	51.7
	30-34	87	20.5
	35-39	55	12.9
	≥40	28	6.6
Gender	Male	258	60.8
	Female	166	39.2
Marital status	Married	171	40.3
	Single	253	59.7
Educational status	Diploma	14	3.3
	Degree and above	410	96.7
Monthly salary	≤5000	165	38.9
	5001-8000	179	42.2
	≥8001	80	18.9
Year of services	< 5 years	295	69.6
	5-10 years	103	24.3
	≥11	26	6.1

Table 2- Behavior, knowledge, and practice related characteristics of midwives working in public health institutions of Addis Ababa city, Addis Ababa, Ethiopia, 2020

Variables	Categories	Frequency	%
Regularly applying universal precautions	Yes	191	45
	No	233	55
Immediate actions following exposure	Washing with soap and water	218	89.3
	Washing with iodine or alcohol solution	29	11.9
	Got tested for HIV	96	39.3
	Took post-exposure prophylaxis (PEP)	42	17.2

Formatted: Header

Variables	Categories	Frequency	%
	Took tetanus anti-toxoid (TAT)	2	0.8
Personal & patient protection	Wear gloves appropriately	362	85.4
	Proper handwashing	301	71
	Wearing gowns and aprons	300	70.8
	Wearing of eye goggles, masks, and shields	276	65.1
Job-related stress	Yes	192	46
	No	232	54
Job satisfaction	Satisfied	176	42
	Dissatisfied	248	58
Ever had tested for HIV/AIDS	Yes	385	90.8
	No	39	9.2
Ever had tested HBV or HCV	Yes	351	79.8
	No	73	20.2
Appropriate hand wash before putting on gloves	Always	150	35.4
	Sometimes	248	58.5
	Never	26	6.1
Hand wash after handling contaminated objects	Always	294	69.3
	Sometimes	125	29.5
	Never	5	1.2
Hand wash after contact with blood or mucous membrane	Always	347	81.8
	Sometimes	69	16.3
	Never	8	1.9
Availability of antiseptic hand rub	Always	142	33.5
	Sometimes	257	60.6
	Never	25	5.9
Use protective barriers appropriately	Always	191	46.7
	Sometimes	223	52.6

Formatted: Header

Variables	Categories	Frequency	%
	Never	10	2.4

Table 3 Predictors of BBFs among midwives working in public health institutions of Addis Ababa city, Ethiopia 2020.

Variables	BBFs		COR(95% CI)	AOR(95% CI)
	Yes=198	No=226		
Gender				
Male	163(84.8%)	95(42%)	6.4(4.1-10.1)	2.1(0.99-4.33)
Female	35(15.2%)	131(58%)	1	
Marital status				
Married	50(25.3%)	121(53.5%)	1	
Unmarried	148(74.7%)	105(46.5%)	3.4(2.3-5.2)	6.02(0.42-10.02)
Did you worked in shift?				
Yes	173(87.4%)	173(76.5%)	2.1(1.26-3.6)	2.36(1.034-5.4)*
No	25(12.6)	53(23.5%)	1	1
Work hours per week				
≥40 hours	158(79.8%)	80(35.4%)	7.2(4.6-11.2)	2.01(0.002-9.03)
≤39 hours	40(20.2%)	146(64.6%)	1	
Receiving IP and safety training in the last year				
Yes	27(13.6%)	184(81.4%)	1	1
No	171(86.4%)	42(18.6%)	27.7(16.4-46.9)	4.27(1.94-9.41)***
Use of safety containers regularly				
Yes	16(8.1%)	171(75.6%)	1	1
No	182(91.9%)	55(24.4%)	35.4(19.5-64.1)	21.01(0.99-26.02)
Regularly applying universal precautions				
Yes	11(5.6%)	180(79.6%)	1	1
No	187(94.4%)	46(20.4%)	66.5(33.4-132.5)	8.43(6.55-14.8)***
Job-related stress				
Yes	151(76.3%)	44(19.5%)	13.3(8.4-21.1)	2.18(1.07-4.49)*
No	47(23.7%)	182(80.5%)	1	1
Year of service				
1-5 years	153(77.3%)	142(62.8%)	2.9(1.2-7.2)	0.23(0.25-2.65)

6-10 years	38(19.2%)	65(28.8%)	1.6(0.6-4.1)	
≥ 10 years	7(3.5%)	19(8.4%)	1	1
Monthly salary category				
≤5000 birr	88(44.4%)	77(34%)	1.26(0.74-2.2)	
5001-8000 birr	72(36.4%)	107(47.3%)	0.74(0.437-1.3)	0.305(0.105-0.88)*
≥8001 birr	38(19.2%)	42(18.7%)	1	1
Job satisfaction				
Satisfied	46(23.2%)	132(58.4%)	1	1
Dis satisfied	152(76.8%)	94(41.6%)	4.6(3-7.1)	3.59(0.87-6.07)

**p=0.04; **p=0.01; ***p=0.001*

Formatted: Header

Author's Point-by-Point Response to the Reviewer's and Editors Reports

Title: Occupational Exposure to Blood/ Body Fluid Splash and Its Predictors among Midwives Working in Public Health Institutions at Addis Ababa City Ethiopia, 2020

Institution-based cross-sectional study

Corresponding author: Solomon Shitu Ayen (solomonsht7@gmail.com)

Authors

Getachew Adugna (getchadu2013@yahoo.com)

Haimanot Abebe (haimanotabebe78@gmail.com)

ID: PONE-D-20-40344
Journal: PLOS ONE
Article type: Research article

First of all, the authors would like to thank PLOS ONE Journal editors and the respective reviewers for reviewing our manuscript and providing the necessary comments to be corrected. As per the comments given, we have made corrections point by point to comment. The authors tried to answer all the issues raised by the editorial team and reviewers. Please note that we gave our response in blue font color.

Point by point response to editor

Comment "Please ensure that your manuscript meets PLOS ONE's style requirements, including those for file naming". And we suggest you thoroughly copyedit your manuscript for language usage, spelling, and grammar

Response 1: Thanks for your great suggestion and timely comments. Corrected

Point by point response to Reviewer# 1

Dear,

Question1; Grammatical edits within the document

Response 1: Thanks for your great suggestion and timely comments. Corrected grammatical errors throughout the document

Question2; Why target this group of health care professionals - not clear from the background

Response 2: Thanks for your great suggestion and timely comments. We included only midwives was as in general little is known about blood and body fluid splash among all health professionals and midwives working more focus on conducting delivery in which they are more susceptible for

fluids and blood splash during attending delivery. Because they are nearest to exposure like amniotic fluid, meconium, blood, vomiting, and so on.

Question3; Rewrite the conclusion

Response 3: Thanks for your great suggestion and timely comments. Corrected as, “The study revealed that nearly half of midwives exposed to BBFS. This sparks light to concerned bodies like policymakers and stack holders to design appropriate policy to avert this magnitude and making the environment enabling to comply with standard precautions. Also, this study was done in urban health settings, and the institutions are better equipped with personal protective materials than the rural settings. So, we recommend that this study may be done by including rural setting institutions and by including other health professionals that are susceptible to BBFS at work. Formal training on infection prevention and safety practice to apply universal precautions will be needed from the concerned bodies to prevent exposures to blood/body fluid splash.”

Question 4; Rewrite the background

Response 4: Thanks for your great suggestion and timely comments. Corrected as Blood/body fluid splash (BBFS) are hazards to health care workers in their working area. Around twenty bloodborne pathogens are known to transmit through this occupational exposure [1]. This problem alters the health status of health care professionals (HCPs) in different ways, including physically, mentally, and psychologically [2].

No occupation or work is free from health hazards. People are always under a certain amount of health risk wherever they are working [3]. Health care professionals are exposed to a wide range of occupational health and safety hazards [4]. An estimated 20 million work-related injuries and 390, 000 new work-related illnesses occur each year in the United States and China 66.3% of HCPs per year exposed to BBFS; however, the number of occupational diseases and injuries reported each year is much more [4, 5].

Nowadays, workplace injuries, especially BBFS prevalence, are increasing among health workers [6, 7]. Occupational exposure to BBFS in healthcare facilities constitutes a significant risk of transmission of human immune deficiency virus (HIV) and other blood-borne pathogens to healthcare workers [8-10]

As estimated by the Center for Disease Control (CDC) that every year more than three million HCPs are exposed to BBFs, through sharps, plus mucocutaneous injuries in the United States [10]. Due to BBFS, the possibility of infections ranges from as low as 0.2–0.5% for HIV to as huge as 3–

10% for Hepatitis C virus (HCV) and 40% for Hepatitis B virus (HBV) [11]. World health organization (WHO) estimates that nearly 3 million HCPs experience occupational exposure to blood-borne viruses HBV, HCV, and HIV has been reported to be 2.1 million, 926,000, and 327,000 respectively [12-15]. From this 90% of this exposure to infection was resulted from low resource countries [14]. In Ethiopia, studies indicated that the prevalence of HCV due to BBFS exposure was 0.9- 5.8% [3, 16].

Health care workers in Africa experience from two to four needle-stick injuries per year on average, with Nigeria, Tanzania, and South Africa reporting on average of 2.10 injuries per HCPs per year [17].

Even though there is no national data regarding the prevalence of BBFS among midwives in Ethiopia previous studies in Eastern, Southern, and Northern parts have shown the increased risk of occupational blood exposure [17-19]. The majority of these studies were only confined to a single institution and workplace [18, 19]. The high rate of HCPs is affected by the exposure [2]. Despite the presence of many possibilities to reduce the burden of occupational exposure to BBFS, it is not yet recognized. Hence this research was carried out on midwives working at public health institutions of Addis Ababa city delivery unit due to they are more exposed and the first line for BBFS.

Question 5; why you focus only on midwives?

Response 5: Thanks for your great suggestion and timely comments. Even though all health professionals are exposed to BBFS, midwives are the first line for fluid and blood exposure. Because obstetrics is a bloody profession and they are the nearby professional. And all health institutions have employed midwives in Addis Ababa city.

Question 6; Study Population

Randomly selected midwives working in public health institutions of Addis Ababa city.

“How was this done?”

Response 6: Thanks for your great suggestion and timely comments. By using a systematic random sampling technique

Question 7; On **s**ampling procedure, we said The registers of all midwives were obtained from the administration of the offices. “Obtaining this list of names does pose some ethical considerations. Need to clarify how the researchers obtained access to this register. Had the midwives provided informed consent for their names and contact details to be released for research purposes?”

Response 7: Thanks for your great suggestion and timely comments. Even though the study has some ethical issues we directly requested the office by letter but because the study has minimal or no harm on them.

Question 8; more detail of process needed on sampling procedure

Response 8: Thanks for your great suggestion and timely comments. Possible correction taken by each institution has their number of allocated midwives based on that we classify proportionally based on total sample size. "Six public hospitals from 12 hospitals and 66 health centers from 112 health centers were selected by simple random sampling technique. The total number of midwives working in Addis Ababa city was 2432 and the number of midwives working in selected public health institutions was 1296. To select study participants from selected health institutions the proportional allocation method (each institution's number of midwives was allocated by proportion) was used for each institution. Study participants who fulfilled the inclusion criteria were selected by systematic random sampling technique until the required number is obtained with a kth value of 3."

Question 9; Data quality control and collection methods

Using an English version questionnaire "is this a limitation? English literacy levels etc"

Response 9: Thanks for your great suggestion and timely comments. This study was related to their job and doesn't need that much fluent speaker or writer of English. The interviewees were minimum of diploma certificate so they can understand the questionnaire.

Question 10; in result part "is this reflective of the midwifery workforce demographic in Ethiopia? If not then why more males completed"

Response 10: Thanks for your great suggestion and timely comments. Your concern was correct midwifery profession was perceived as a profession of women in the previous era but in the current situation this is changed and more males are involved as midwife professionals. That may be the reason that in this study the percentage of males was greater than females.

Question 11; These data are not presented in a table - sentence structure needs review to make results clear. Also review terminology around HIV/AIDS - was 12.5 living with HIV and been diagnosed with an AIDS-defining illness or is it more accurate to say 12.5% were HIV positive or known to be living with HIV.

Response 11: Thanks for your great suggestion and timely comments. Corrected

Question 12; the meaning of working in shifts is unclear - please clarify

Response 12: Thanks for your great suggestion and timely comments. Corrected as working in two shifts >12 hours work or day and night is more likely exposed than working in only one shift (working for ≤12 hours)

Point by point response to Reviewer# 2

Dear, Yalemzewod Gelaw (PhD)

Question1; I would like to suggest the authors include a justification why they are interested only in midwives and how the risk of exposure to blood/body fluid splash is varied across health professionals supported with prevalence figures. In the context of the Ethiopian health system, all health facilities have no midwives. Therefore, still, other health professionals such as nurses/public health officers/doctors are at risk for exposure.

Response 1: Thanks for your great suggestion and timely comments. We included only midwives was as in general little is known about blood and body fluid splash among all health professionals and midwives working more focus on conducting delivery in which they are more susceptible for fluids and blood splash during attending delivery. Because they are nearest to exposure like amniotic fluid, meconium, blood, vomiting, and so on. Since the study was done in Addis Ababa city the work of the delivery unit was covered by midwives in all health institutions even if the number of professionals is not equal to the standard.

Question 2; "Study setting – the risk of exposure could be high in health professionals working in rural settings than in health professionals working in urban. It would be great if the authors could consider the effect of health facilities setting".

Response 2: Thanks for your great suggestion and timely comments. This is a correct issue that the study was better sound than this if it included the rural setting but due to different reasons, it was done only in the urban area even though little was known on the topic. We recommend other researchers incorporate rural settings in future studies.

Question 3; Source Population – does the result will be generalized only to those midwives working in AA? "All midwives working in public health institutions of Addis Ababa city'' – is the population where your sample was drawn i.e., study population unless the authors' hypothesis was only for urban midwives.

Response 3: Thanks for your great suggestion and timely comments. Yes, this study was generalized for urban setting midwives. Because the source population was midwives working in public health institutions of Addis Ababa city

Question 4; Inclusion criteria – is there unregistered midwives working in the current Ethiopian health system? I would suggest the authors modify this.

Response 4: Thanks for your great suggestion and timely comments. Corrected as "All midwives working in Addis Ababa city"

Question 5; Sample size determination – I would recommend the authors to include the sample size assumptions briefly such as proportion from the previous study, margin of error, etc.

Response 5: Thanks for your great suggestion and timely comments. Corrected as "A separate sample size was calculated for each specific objective by using both single and double population proportion formula was used. For the first specific objective, (To determine the prevalence of occupational exposure to BBFS among midwives working in Addis Ababa city) single population proportion was used.

$n = \frac{(Z\,\alpha/2)^2 P\,(1-P)}{d^2}$ *by the* assumption of n= minimum sample size required for the study

$(Z\,\alpha/2)^2$ = standard normal distribution with 95% confidence interval, P= 21% (prevalence of occupational exposure and behavior of health care workers in Ethiopia) [18], d= is a tolerable margin of error (d=0.04)

The sample size of the first objective was greater than that of the second objective. By adding a 10% non-response rate from the largest sample size the calculated sample size was 438."

Question 6; Sampling procedures – "…. simple random sampling technique until the required number is obtained." For simple random sampling techniques, all eligible study participants should be identified before the data collection starts. Including study participants, until the required number is obtained will work for systematic sampling techniques.

Response 6: Thanks for your great suggestion and timely comments. Corrected as "The total number of midwives working in Addis Ababa city public health institutions was 2432 and number of midwives working in selected institutions was 1296. To select study participants from selected health institutions the proportional allocation method (each institution's number of midwives was allocated by proportion) was used. Study participants who fulfilled the

inclusion criteria were selected by systematic random sampling technique until the required number is obtained with a kth value of 3”.

Question 7; The independent variables except the demographic variables need to be clearly defined.

Response 7: Thanks for your great suggestion and timely comments. Corrected as Work-related factors: (Training on IP, working environment, year of service, educational level)

Behavioral factors: (Job satisfaction, reporting after the exposure, using the universal precautions and personal protective equipment)

Question 8; I would like to ask the authors to use variables with the (P-value < 0.25) to include in the multivariable analysis. For the authors' reference – to read applied logistic regression book by Hosmer and Lemeshow.

Response 8: Thanks for your great suggestion and timely comments. Corrected as “The collected data were checked and reviewed for completeness, coded, and entered into Epi data version 3.1 and exported to SPSS version 24 for analysis. Descriptive statistics were used to determine the frequency of different variables. Association between dependent and independent variables were examined using bi-variable and multivariable logistic regression models. Those variables which were (P-value < 0.25) [18] in the bivariate analysis were included in the final model of multivariate analysis to control all possible confounders. Multi-collinearity was checked to see the linear correlation among the independent variables by using standard error. Variables with a standard error of > 2 were dropped from the multivariable analysis. Model fitness was checked with the Hosmer-Lemeshow test. The direction and strength of statistical association were measured by the odds ratio with 95 % CI using multivariable logistic regression analysis. Adjusted odds ratio along with 95% CI was estimated to identify factors associated with BBFS. In this study P-value < 0.05 was considered to declare a result as statistically significant”.

Question 9; Socio-demographic characteristics – report median age instead of the mean. The min and max age showed the distribution is not normal.

Response 9: Thanks for your great suggestion and timely comments. Corrected as “The age distribution of the respondents ranges from 20-56 years, with a mean and median age of 30.11 (SD ± 5.37), 26.5 years respectively and 219 (51.7%) were between the age group of 25-29 years”.

Question 10; Regression output – interpret only the multivariable results.

Response 10: Thanks for your great suggestion and timely comments. Corrected as “In multivariable logistic regression working in >12 hours shift, not receive IP and safety practice training, not regularly apply universal precautions, job-related stress, an average monthly salary of 5001-8000 were found independent predictors of BBFS.

Health care professionals who did not regularly apply universal precautions were 8 times more likely to have blood/body fluid splash (AOR 8, 95% CI 6.55 - 14.8) than their counterparts. Participants who did not receive IP and safety practice training were four times more likely to have blood/body fluid splash (AOR 4.27, 95% CI 1.94 - 9.41) than those who did receive training. The odds ratio was two times higher for those working in two shifts (more than 12 hours) (AOR 2.36, 95% CI 1.034-5.4) than one shift (≤ 12 hours). The odds of exposure to BBFs were two times increased for those who had job-related stress (AOR 2.18, 95% CI 1.07-4.49) than their counterpart. Respondents who get an average monthly salary of 5001-8000 Ethiopian birr were 70% less likely to be exposed to BBFS (AOR 0.305, 95% CI 0.105 - 0.88) than those who had gained less than 8000 Ethiopian birrs.

Question 11; Discussion section is very weak and needs to be improved.

Response 11: Thanks for your great suggestion and timely comments. Rewritten and improved as much as possible
