# Supplementary material for: Occupational exposure to blood/body fluid splash and its predictors among midwives working in public health institutions at Addis Ababa city Ethiopia, 2020. Institution-based cross-sectional study
Source: PLoS One. 2021 Jun 18;16(6):e0251815. doi: 10.1371/journal.pone.0251815 (PMC8213160; doi:10.1371/journal.pone.0251815)
Supplement: Attachment — Submitted filename: Response to Reviewers.docx [file pone.0251815.s006.docx]

**Author’s Point-by-Point Response to the Reviewer's and Editors Reports**

# **Title**: Occupational Exposure to Blood/ Body Fluid Splash and Its Predictors among Midwives Working in Public Health Institutions at Addis Ababa City Ethiopia, 2020

Institution-based cross-sectional study

**Corresponding author**: Solomon Shitu Ayen ([solomonsht7@gmail.com](mailto:solomonsht7@gmail.com))

**Authors**

Getachew Adugna	([getchadu2013@yahoo.com](mailto:getchadu2013@yahoo.com))

Haimanot Abebe	(haimanotabebe78@gmail.com)

**ID**: PONE-D-20-40344

**Journal**: PLOS ONE

**Article type:** Research article

First of all, the authors would like to thank PLOS ONE Journal editors and the respective reviewers for reviewing our manuscript and providing the necessary comments to be corrected. As per the comments given, we have made corrections point by point to comment. The authors tried to answer all the issues raised by the editorial team and reviewers. Please note that we gave our response in blue font color.

**Point by point response to editor**

Comment “We recommend that you carefully consider the comments from reviewer 1 in your revision”.

**Response 1**: Thanks for your great suggestion and timely comments. Corrected

**Point by point response to Reviewer# 1**

Dear,

**Question1;** comment on conclusion

**Response 1**: Thanks for your great suggestion and timely comments. Corrected as you recommendation

**Question 2;** the paper still requires a thorough edit of language

**Response 2**: Thanks for your great suggestion and timely comments. We have taken corrections concerning to any English errors and typos by the help of a man who have PhD in English language. Furthermore, we have also taken correction for any English errors using online grammar and English typo correctors apps (We used the following links-

[https://app.grammarly.com/?network=g&utm_source=google&matchtype=e&gclid=Cj0KCQjwo-aCBhC-ARIsAAkNQiuJ49UHhl6ibhQfzq9D4wGrbSOeZPv49UoRqSnd4ThQ-KKrPp uBp4aAjLgEALw_wcB&placement=&q=brand&utm_content=486649398671&gclsrc=aw.ds&utm_campaign=brand_f1&utm_medium=cpc&utm_term=grammarly](https://app.grammarly.com/?network=g&utm_source=google&matchtype=e&gclid=Cj0KCQjwo-aCBhC-ARIsAAkNQiuJ49UHhl6ibhQfzq9D4wGrbSOeZPv49UoRqSnd4ThQ-KKrPp%20uBp4aAjLgEALw_wcB&placement=&q=brand&utm_content=486649398671&gclsrc=aw.ds&utm_campaign=brand_f1&utm_medium=cpc&utm_term=grammarly) and <https://pubsure.researcher.life/author/?active_tab=recent_plan>

So, now we have solved all iniquities related with the English language including the tense used and unnecessary capitalization and other typos/ errors

**Question 3;** relevance to paper - Introduction needs further refining to more clearly link t rationale for why Midwives are considered high risk - group. More information is added to the responses to the reviewer’s queries that could strengthen the paper if inserted into the text body

**Response 3**: Thanks for your great suggestion and timely comments. Corrected as you recommendation

**Question 4;** permission for what ? Is this authoriastion to access the register of midwives. This needs to be more specific and clear that this is in line with ethical standards of practice as this sharing of personal details of registered staff would not be allowed in many context under Pravacy acts. .

**Response 4**: Thanks for your great suggestion and timely comments. The formal letter submitted to the institutions was used as to ask permission and to show the legal step that we go while conducting this study.

**Question 5;** what is the difference between written and signed

**Response 5**: Thanks for your great suggestion and timely comments. Written and signed means we or data collectors prepare written document that the participants sign on it.

Corrected as “An official letter of permission was submitted to the Addis Ababa city health bureau. A formal letter was submitted to selected public health institutions from the bureau. The respondents were informed about the objective, purpose, risks, and benefits of the study and the right to refuse to participate, and all subjects provided informed written consent for inclusion before they participated in the study”.

**Question 6;** The status of the source of exposure was unknown for 79 (39.5%), whereas, 25(12.5%) were known HIV positive, 16 (8%) were clinically suspected HIV positive, however, 77(38.5%) known HIV negative. “sentence structure needs review”

**Response 6**: Thanks for your great suggestion and timely comments. Corrected as “From those who were exposed, the status of the source of exposure was unknown for 79 (39.5%), whereas, 25(12.5%) were known HIV positive and 16 (8%) were clinically suspected HIV positive. However, 77(38.5%) known HIV negative.”

**Question 7;** discussion “sentence structure - make prevalence of what clear”

**Response 7**: Thanks for your great suggestion and timely comments. Corrected as “In this study, the prevalence of BBFS at least once in their work life was 198 (46.7%) with 95%CI: 41.3-52.2.”

**Question 8;** discussion “was this Australian study involving midwives or who. Same for the other studies cited - need to make sure you are comparing similar groups and if not then discuss what this means”

**Response 8**: Thanks for your great suggestion and timely comments. There is no study done only on midwives but we used literatures done on BBFS. So we discussed with those literatures done on BBFS.

**Question 9;** Do you mean midwives who worked across two shifts that is > 12 hours - for the international reader you need to make clear what normal shift working hours are and whether these long hours are the norm so therefore a common risk for midwives. I think it is clearer if you just acknowledge the <12 and >12 hours and not focus on the shifts

**Response 9**: Thanks for your great suggestion and timely comments. Corrected

**Question 10;** limitation And the study has done only on midwives so better to include other health professionals “sentence structure”

**Response 10**: Thanks for your great suggestion and timely comments. Corrected as “The study was done only on midwives so the study will sound better if it includes other health care professionals.”
